# Host Genetic Architecture between Epstein–Barr Virus Activity and Multiple Sclerosis Reveals Shared Pathways

**DOI:** 10.64898/2025.12.11.25342083

**Published:** 2025-12-15

**Authors:** Yoshiaki Yasumizu, Namkwon Kim, Cyprien A. Rivier, Jeonghyeon Moon, Shohei Kojima, Heng-Le Chen, Nicholas Buitrago-Pocasangre, Elizabeth Quinn, Stephen Vaughn, Annalisa Morgan, Shufan Huo, Andrew Silberfeld, Tomokazu S. Sumida, Kazuyoshi Ishigaki, Erin E. Longbrake, Guido J. Falcone, David A. Hafler

**Affiliations:** 1 Department of Neurology, Yale School of Medicine, New Haven, CT, 06510, USA; 2 Yale Center for Brain and Mind Health, Yale School of Medicine, New Haven, CT, 06510, USA; 3 Keio University Human Biology-Microbiome-Quantum Research Center (WPI-Bio2Q), Tokyo, 160-8582, Japan; 4 Department of Microbiology and Immunology, Keio University School of Medicine, Tokyo, 160-8582, Japan; 5 Laboratory for Human Immunogenetics, Riken Center for Integrative Medical Sciences, Kanagawa, 230-0045, Japan; 6 Department of Immunobiology, Yale School of Medicine, New Haven, CT, 06510, USA; 7 Broad Institute of MIT and Harvard, Cambridge, MA, 02142, USA

## Abstract

Epstein–Barr virus (EBV) is strongly implicated as an essential environmental trigger of multiple sclerosis (MS), yet the host genetic mechanisms governing EBV activity and how infection triggers the disease are not known. We developed a pipeline to quantify EBV DNA from whole-genome sequencing data and applied it to population-scale cohorts. Using this pipeline, we performed a cross-ancestry genome-wide association study (GWAS) of EBV DNA positivity in 617,186 individuals and identified 39 independent susceptibility risk loci, with the strongest associations in the HLA region. We validated this finding in our independent cohort (*N*=94) and found that quantitative PCR (qPCR)-confirmed EBV DNA positive individuals were enriched in the top decile of EBV polygenic risk scores (PRS) containing newly discovered loci. A significant overlap with genetic variants associated with MS risk was observed. PRS and Mendelian randomization analyses further supported a causal role of EBV activity on MS risk, which was also seen in other autoimmune diseases. A meta-analysis of qPCR based case–control studies showed elevated EBV DNA positivity in MS. By establishing a single-cell RNA-seq method optimized for EBV detection, we identified EBV-infected B cells, primarily in memory B cells, atypical B cells and antibody-secreting cells from MS and healthy individuals. Notably, EBV-infected memory B cells and atypical B cells showed strong upregulation of cytokines and costimulatory signals that influence T cell activation, IFNg secreting Tregs, and regulators of B cell differentiation and survival. EBV-infected memory B cells also upregulated risk genes from both the EBV and MS GWAS, suggesting that EBV-infected B cells constitute a critical hub that modulates T cell responses while simultaneously activating MS susceptibility pathways within the B cell compartment. Together, these findings define a genetic and cellular framework linking EBV infection to the initiation of MS.

## Introduction

Multiple sclerosis (MS) is the most common central nervous system (CNS) disease of young adults. The disease most commonly begins with an autoimmune, relapsing remitting phase mediated by circulating autoreactive T cells^1^ that migrate to the central nervous system (CNS) and induce a marked inflammatory response both in the white matter and cortical grey matter. A secondary, neurodegenerative disease course occurs in a significant number of patients, presumably triggered by the initial T cell-mediated autoimmune process. As with other autoimmune disorders, MS is triggered by unfavorable environmental interactions in genetically susceptible individuals. Indeed, we have identified 233 common genome loci associated with disease risk and one variant associated with disease progression.^2–4^ The majority of these map to the immune system, targeting T cells, regulatory T cells, and, in particular, B cells.^2,5,6^ As a result, MS patients exhibit a spectrum of immunopathology, including a skewing of T cells toward pro-inflammatory Th1 and Th17 programs,^6–8^ loss of regulatory T-cell function,^9,10^ and activation of B cells.^11^

Compelling epidemiologic data implicates Epstein–Barr virus (EBV) as the initiating event breaking immune tolerance in genetically susceptible individuals. A large longitudinal study of U.S. military personnel reported that EBV infection occurs before MS development^12^, indicating that EBV is a necessary causal factor for MS. Serum neurofilament light chain, a marker of axon injury, begins to rise only after EBV seroconversion. Moreover, MS patients exhibit higher EBV activity,^13–15^ increased frequencies of EBV-reactive T cells^16,17^, and EBV-derived antigens that cross-react with myelin antigens^18,19^. EBV primarily infects B cells, directly influencing their function^20^, and we have observed distinctive alterations in B-cell phenotypes in MS (*manuscript in preparation*). Importantly, B-cell depletion therapy is a highly effective treatment for the early relapsing remitting, autoimmune phase of MS,^21^ underscoring the central role of B cells in disease pathogenesis.

EBV plays a causal role in several other human diseases. Epidemiologic and immunologic evidence links EBV to non-Hodgkin lymphoma, including Burkitt lymphoma, nasopharyngeal carcinoma, gastric carcinoma, and, as discussed above, to MS, rheumatoid arthritis (RA), and systemic lupus erythematosus (SLE).^22^ Although EBV infection is ubiquitous affecting more than 90% of the global population, its clinical manifestations vary widely. In most individuals, EBV infection remains asymptomatic or latent, whereas a subset of subjects develop infectious mononucleosis or chronic active EBV infection. Moreover, infectious mononucleosis is not only a symptomatic form of EBV infection but also a strong epidemiological risk factor for MS.^23^ However, few individuals develop malignancies or autoimmune diseases after EBV infection. This variability suggests that the host’s ability to control EBV reactivation differs among individuals, potentially due to underlying genetic factors.^24^

EBV activity can be assessed through two major biomarkers: anti-EBV antibodies and EBV DNA levels. Immunoglobulin (Ig) G targeting EBV antigens reflects prior exposure. Over 90% of healthy adults and nearly 100% of patients with MS are seropositive.^15,25^ In contrast, EBV DNA directly measures viral activity; blood EBV DNA is increased in patients with MS and SLE.^14,26^ Circulating EBV DNA is also clinically valuable for nasopharyngeal carcinoma screening.^27^ In aggregate, these data suggest that circulating EBV DNA load would be a novel tool to investigate viral activity as linked to the genetic background.

Genome-wide association studies (GWAS) have allowed identification of genetic variants associated with various traits such as diseases or viral infections. For example, measurement of HIV viral load overcame the limited resolution of infection-status GWAS and successfully identified key loci in the HLA and *CCR5* regions.^28^ Recent advances in sequencing technologies now allow simultaneous quantification of host and viral genomes from the same dataset. Our group previously developed *VIRTUS*,^29^ a pipeline for detecting viral transcripts from RNA-seq data, and EBV detection from bulk RNA-seq has also been reported by others.^30^ Similar approaches have recently been applied to whole-genome sequencing (WGS) data, leading to a GWAS of human herpesvirus 6 (HHV-6) viral load that revealed integration sites and links to SLE disease activity.^31^ For EBV, however, prior GWAS have focused almost exclusively on serologic antibody titers,^32^ which capture infection history rather than active viral replication. Only one prior EBV DNA GWAS, conducted in 10,585 Chinese participants, has been published;^33^ limited sample sizes precluded the identification of genome-wide significant associations.

**Extended Data Figure 1:**
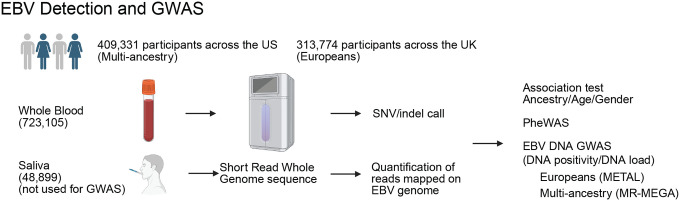
Study design of cross-ancestry meta-analysis of EBV DNA GWAS.

Here, we analyzed 617,186 participants from the All of Us Research Program^34^ and the UK Biobank,^35^ applying harmonized viral-detection and quality-control pipelines across ancestries. By performing meta-analysis across these cohorts, we effectively increased the sample size, enabling the largest EBV DNA GWAS (N=617,186) to date (Extended Data Fig. 1). We further integrated phenome-wide association studies (PheWAS), Mendelian randomization (MR), and polygenic risk score (PRS) analysis to systematically assess the causal relationships between EBV activity and human diseases. Combining GWAS with single-cell RNA-seq (scRNAseq) and spatial transcriptomic analyses, we identified specific cell types and anatomical niches involved in EBV control. Notably, using *VIRTUS3*, we successfully identified EBV-infected cells from people with MS and characterized their genetic effects.

Together, these findings provide a large-scale, multi-ancestry genetic map of host control over EBV activity and EBV infection and transcriptome dynamics in people with MS at single-cell resolution, offering mechanistic insight into EBV-associated autoimmunity, establishing a foundation for the discovery of therapeutic targets aimed at modulating EBV reactivation, and elucidating a mechanism by which genetic variants associated with MS risk drive EBV infection in B cells, potentially breaking immune tolerance.

## Results

### EBV DNA detection from WGS

**Figure 1:**
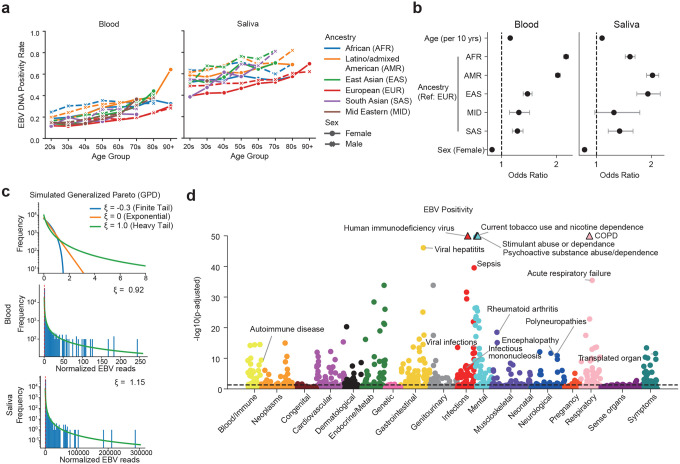
EBV DNA characteristics (a) EBV DNA positivity across predicted ancestors, gender, and age in blood (left) and saliva (right). (b) Forest plots showing odds ratios (points) and 95% confidence intervals (horizontal lines) from logistic regression of EBV DNA positivity for blood samples (left) and saliva samples (right). The age effect represents the change per 10-year increase. European ancestry and male sex were used as references. The dashed line indicates *Odds ratio (OR)*=1. (c) Distribution of EBV DNA fitted with Generalized Pareto distribution (GPD). Left: Simulated examples of GPD probability density functions illustrating the effect of the shape parameter ξ on the tail behavior: finite tail (ξ < 0), exponential tail (ξ = 0), and heavy tail (ξ > 0). Middle and Right: Histograms of normalized EBV DNA reads in blood (middle) and saliva (right). Orange curves represent GPD fits above the 95th percentile threshold (red dashed line). Estimated ξ values are shown for each sample type. (d) Phenome-wide association study (PheWAS) of EBV DNA positivity in European participants from the All of Us cohort. Each point represents a clinical phenotype, colored by disease category. The x-axis denotes phenotype groups and the y-axis shows the *–log*_10_*(FDR-adjusted P value)*. The dashed line indicates the FDR threshold of 0.05. Details are available in Supplementary Table 1. AFR, African ancestry; AMR, Latino/admixed American ancestry; EAS, East Asian ancestry; EUR, European ancestry; SAS, South Asian ancestry

To detect EBV reads in WGS datasets, we developed an analysis pipeline that filters and counts reads mapped to the EBV decoy sequence in the human reference genome. We applied our pipeline to the diverse cohort of study participants enrolled in the All of Us Research Program. After quality control, 409,331 individuals were included. DNA information was obtained from two sources: whole blood (*N*=359,796) and saliva (*N*=48,086). EBV DNA was detected in 20.8% of blood samples and 52.7% of saliva samples. Age, sex, and ancestry were significantly associated with EBV positivity. In both blood and saliva, higher age was strongly associated with higher detection rates (Fig. 1a, blood: *OR*=1.16 per 10 years, 95% *CI*=1.15–1.16, *p*<1×10^−100^; saliva: *OR*=1.10 per 10 years, *95% CI*=1.09–1.11, *p*<1×10^−65^). Female sex was associated with a lower rate of EBV positivity compared with males (Fig. 1a, blood: *OR*=0.83, *95% CI*=0.81–0.84, *p*<1×10^−100^; saliva: *OR*=0.78, *95% CI*=0.75–0.81, *p*<1×10^−35^).

When examining ancestry, Europeans had the lowest EBV positivity, whereas all other ancestries showed significantly higher odd ratios of EBV expression. In blood, African (*OR*=2.17, *p*<1×10^−100^), Latino/admixed American (*OR*=2.02, *p*<1×10^−100^), East Asian (*OR*=1.47, *p*=9.2×10^−43^), Middle Eastern (*OR*=1.31, *p*=1.2×10^−4^), and South Asians (*OR*=1.29, *p*=6.1×10^−11^) ancestries had significantly higher odds ratios relative to Europeans (Fig. 1b). In saliva, African (*OR*=1.61, *p*=4.1×10^−65^), Latino/admixed American (*OR*=2.02, *p*<1×10^−100^), East Asian (*OR*=1.94, *p*=5.8×10^−32^) and South Asian ancestries also showed increased odds (*OR*=1.42, *p*=1.4×10^−5^) (Fig. 1b). Middle Eastern ancestry showed a trend for higher EBV positivity in saliva but did not reach statistical significance (*OR*=1.32, *p*=0.078).

Next, we examined the distribution of EBV DNA load. When normalized by the number of reads mapped to the human genome, the EBV DNA load was on average 3,826-fold higher in saliva compared to blood (Fig. 1c). Moreover, a subset of individuals carried viral loads that far exceeded the population average. Indeed, in blood, the top 1% of samples accounted for 64% of the total EBV DNA load, with the top 5% and 10% contributing 83% and 91%, respectively. In saliva, the skew was even more pronounced: the top 1% accounted for 80%, the top 5% for 94%, and the top 10% for 97%. To statistically evaluate this trend, we approximated the distribution with a generalized Pareto distribution (GPD). A positive shape parameter (ξ) indicates a heavy-tailed distribution (Fig. 1c). In blood, *ξ* was estimated as 0.92 (95% *CI*:0.86–0.98) at the 95th percentile threshold, while in saliva, *ξ* was even larger at 1.15 (95% *CI*:1.04–1.25). Varying the threshold from the 90th to 99th percentiles yielded consistently positive *ξ* values, confirming the robustness of these results. These findings demonstrate that the distribution of EBV DNA load is heavy-tailed in both blood and saliva, suggesting the presence of a small number of super-carriers who contribute disproportionately to the overall viral burden.

**Extended Data Figure 2:**
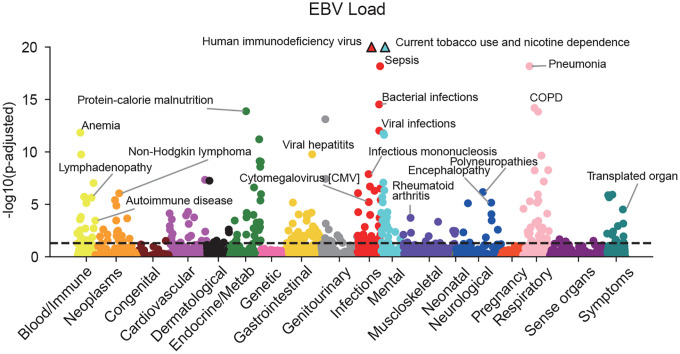
Phenome-wide association study (PheWAS) of DNA EBV viral load in European participants from the All of Us cohort. Phenome-wide association study (PheWAS) of normalized EBV DNA load in European participants from the All of Us cohort. Each point represents a clinical phenotype, colored by disease category. The x-axis denotes phenotype groups, and the y-axis shows the *−log*_10_*(FDR)*. The dashed line indicates the FDR threshold of 0.05. Details are available in Supplementary Table 2.

To comprehensively investigate diseases co-occurring with EBV infection, we conducted a Phenome-wide Association Study (PheWAS). Using European-ancestry participants from All of Us, we performed a PheWAS of EBV DNA positivity and viral load (Fig. 1d and Extended Data Fig.2, Supplementary Tables 1 and 2). Both EBV DNA positivity and viral load showed strong associations with immunosuppressive conditions such as HIV infection, post-transplant immunosuppression, and malnutrition. Notably, there were also strong associations with tobacco use and chronic obstructive pulmonary disease (COPD).^36^ Furthermore, the analysis identified a significant association with infectious mononucleosis, a manifestation of primary EBV infection in adolescents. Unexpectedly, neither MS nor SLE emerged as positively associated traits (MS: EBV positivity, *OR*=0.83, *FDR*=0.034; viral load, *FDR*=0.78. SLE: EBV positivity, *FDR*=0.27; viral load, *FDR*=0.96). In addition to confirming known associations with rheumatoid arthritis, we observed correlations with encephalitis, encephalopathy, and polyneuropathies, suggesting links to other inflammatory neurological diseases. Although more traits were significantly associated with EBV DNA positivity (*FDR*<0.05, 475 traits) than with EBV viral load (270 traits), the viral load analysis specifically highlighted associations with virus-related malignancies such as non-Hodgkin lymphoma and T cell lymphoma, as well as myopathy. The PheWAS results not only reproduced well-established associations between EBV and related diseases, validating our analytical pipeline, but also revealed broader links to autoimmune and inflammatory disorders, underscoring the extensive disease spectrum influenced by EBV infection.

### Multi-Ancestry genome-wide association study of EBV DNA

**Figure 2:**
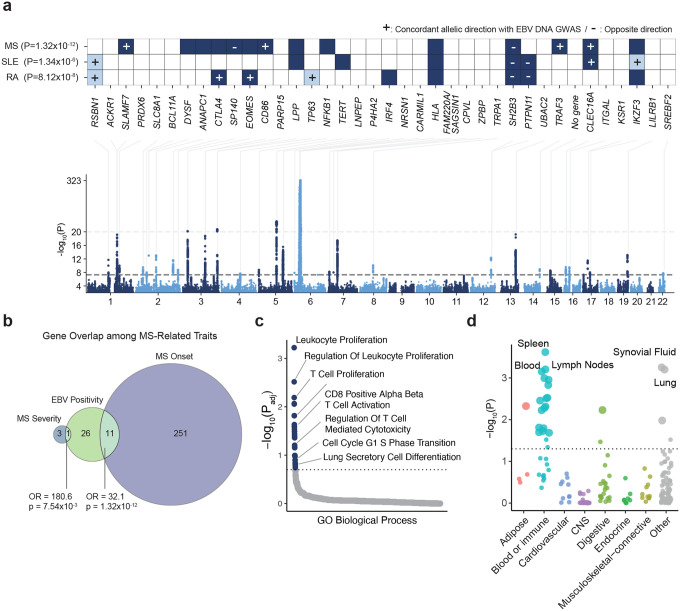
EBV DNA GWAS (a) Manhattan plots showing genome-wide association statistics of EBV DNA expression. The y-axis shows −log_10_P of each variant, and lead SNPs and annotated genes were highlighted. Heatmap shows the genes previously reported in MS,^2,4,103^ SLE,^104^ and RA^105^ GWAS. The dashed line indicates a genome-wide significant level (*p*=5.0×10^−8^). Symbols indicate effect direction relative to the EBV GWAS (+ concordant, − discordant). Directional symbols are displayed only for loci reaching *P*<5×10^−^ in the respective MS/SLE/RA GWAS, while nonsignificant or unavailable associations are shown as blank cells. Genes not reported in the GWASs with *P*<5×10^−^ in the most significant loci of EBV DNA positivity GWAS were highlighted in light blue. Details are also available in Table S4. (b) Overlap of genes highlighted in MS onset GWAS,^2^ MS severity GWAS^4^ and EBV positivity GWAS. Statistical significance was evaluated using a two-tailed Fisher’s exact test on a 2×2 contingency table, with all protein-coding genes (Gencode v47) used as the background (a and b). (c) Gene ontology biological process pathways enriched in GWAS of EBV DNA Positivity. (d) Partitioned heritability was estimated using LD score regression for EBV positivity. Each dot represents a tissue or cell-type annotation, and the size indicates whether the enrichment passed the FDR < 0.2 threshold.

We applied a similar pipeline to the UK Biobank, in which WGS was performed using DNA derived from whole blood. EBV-derived DNA was detected in 15.5% of UK Biobank samples. We then performed a cross-ancestry GWAS combining UK Biobank samples with blood-derived samples from the All of Us cohort. We selected 5,020,877 SNPs with a minor allele frequency (MAF) of ≥1% in All of Us (Methods). Association tests were performed using Regenie^37^ in all ancestry groups from the All of Us Research Program and in European participants from the UK Biobank. A European meta-analysis was conducted using METAL^38^ (N=493,312), and a cross-ancestry meta-analysis (European from UK Biobank and European, African, and Latino/Admixed American from All of Us) was performed using MR-MEGA^39^ (N=617,408) (Supplementary Table 3). In the cross-ancestry analysis, we identified 39 genome-wide significant loci associated with EBV positivity and 16 loci associated with viral load (Fig. 2a and Extended Data Fig. 4a, Supplementary Tables 4–6). Using LD score regression (LDSC),^40^ the SNP-based heritability (h2) of EBV positivity was estimated at 1.77% (*SE*=0.22%), while that of viral load was 1.24% (*SE*=0.18%). There was a positive genetic correlation between EBV positivity and EBV viral load (*rg*=0.71, *p*=4.08×10^−24^).

**Extended Data Figure 3:**
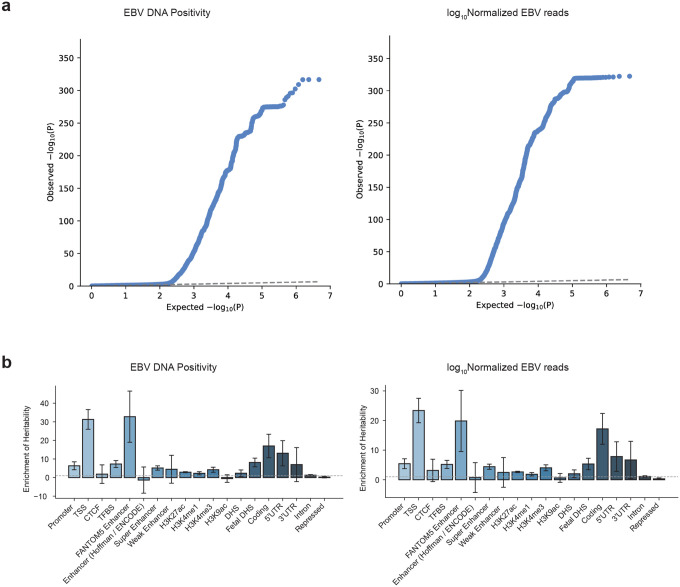
QC plots and annotation of GWAS hits. (a) QQ plot of GWAS for EBV DNA positivity (left) and EBV DNA load (right). (b) Partitioned heritability enrichment in various genomic categories calculated by S-LDSC. EBV DNA positivity (left) and EBV DNA load (right).

We observed a slight inflation of test statistics in the cross-ancestry meta-analysis, particularly for the binary trait (Extended Data Fig. 3a, EBV positivity, *λ*_*GC*_=1.15; viral load, *λ*_*GC*_=1.07). Importantly, the *λ*_*GC*_ values from each individual GWAS were well controlled (*λ*_*GC*_<1.08 in all cases, Supplementary Table 3). We evaluated whether the MHC contributed to the inflation; removing this region yielded comparable values (EBV positivity, *λ*_*GC*_=1.14; viral load, *λ*_*GC*_=1.06). Of note, in the LDSC analysis, intercepts were low (EBV positivity, *intercept*=1.04; viral load, *intercept*=1.03), indicating that the observed inflation is likely due to polygenicity rather than residual confounding.

**Extended Data Figure 4:**
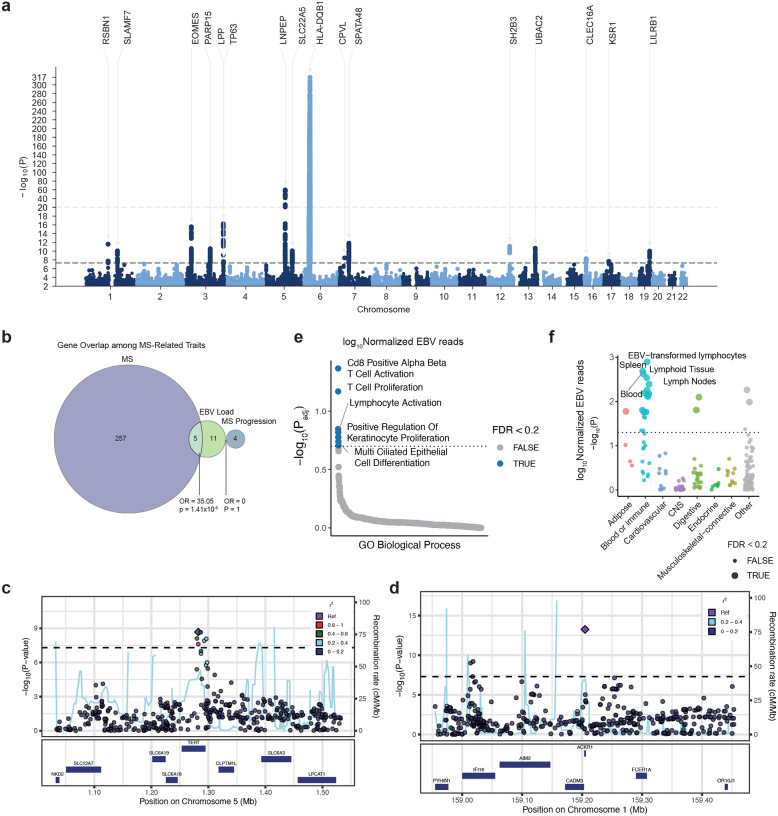
Supportive data for GWAS. (a) Manhattan plots showing genome-wide association statistics of EBV DNA load. log_10_(Normalized EBV read) The y-axis shows −*log*_*10*_*P* of each variant, and lead SNPs and annotated genes were highlighted. The dashed line indicates a genome-wide significant level (*p*=5.0×10−8). Details are also available in Supplementary Table 4. (b) Overlap of genes highlighted in MS onset GWAS, MS severity GWAS, and EBV load GWAS. Statistical significance was assessed using a two-tailed Fisher’s exact test. (c and d) Locusplot of rs7726159 (*TERT2*) and rs2814778 (*ACKR1*, located near *IFI16*) in EBV Positivity GWAS (e) Gene ontology biological process pathways enriched in GWAS of EBV DNA Load. (f) Partitioned heritability was estimated using LD score regression for EBV DNA load. Each dot represents a tissue or cell-type annotation, and the size indicates whether the enrichment passed the *FDR*<0.2 threshold.

**Figure 3:**
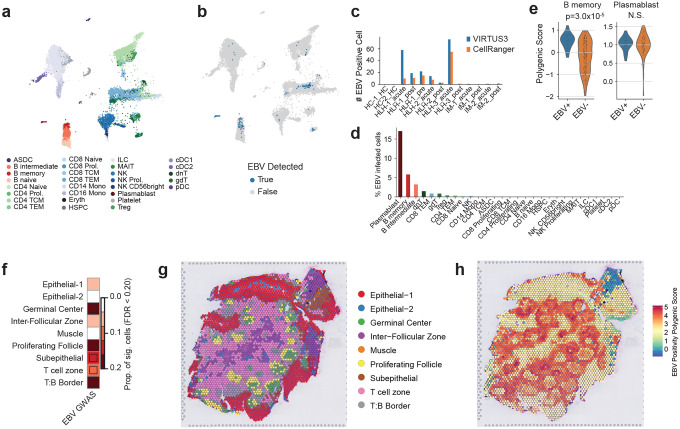
Tissue- and cell-type–specific heritability enrichment for EBV positivity and viral load. (a) UMAP visualization of PBMC single-cell RNA-seq data from EBV-positive and control individuals (DRA017005),^49^ showing major immune cell clusters. (b) Cells with detected EBV transcripts identified by VIRTUS3 (blue) overlaid on the UMAP plot. (c) Comparison of the number of EBV-positive cells detected by VIRTUS3 versus Cell Ranger across samples. (d) Distribution of predicted cell types among EBV-positive cells, highlighting enrichment in plasmablasts and memory B cells. (e) Polygenic scores derived from the EBV DNA positivity GWAS in B memory and plasmablast populations. The Mann-Whitney U test was applied. (f) Region-level enrichment of EBV DNA positivity polygenic signals in tonsils^102^ estimated by scDRS-spatial. Heatmap colors depict the proportion of significant cells (*FDR*<0.2) evaluated using scDRS^48^ Squares denote significant disease associations (*FDR*<0.05). (g and h) Spatial transcriptomic map of human tonsil tissue showing annotated microanatomical regions and EBV DNA positivity polygenic scores across the same tissue section.

Next, we examined the lead SNPs within each locus in detail. Outside of the HLA region, approximately half of the associated variants were intronic (Supplementary Table 4, positivity:63.1%; viral load:53.3%), and roughly half were located in cis-eQTLs (Supplementary Table 4, positivity:55%; viral load:60%). The majority of these loci (Supplementary Table 4, positivity: 65.8%; viral load: 60%) have been previously implicated in immune-related traits, including MS, SLE, RA, IgM levels, and blood cell counts in prior GWAS (Fig. 2a). Remarkably, there was a significant overlap with genetic variants associated with MS risk (Fig. 2B, *OR*=32.1, *p*=1.3×10^−12^) and severity (Fig. 2B, *OR*=180.6, *p*=7.54×10^−3^) including MS susceptibility alleles, HLA-DRB1*04:04, HLA-A*02:01 and to a lesser extent, *HLA-DRB*1*15:01. Specifically, among the top hits, 29.7% of the EBV positivity-associated genes and 33% of the viral load-associated genes overlapped with genes reported in MS GWAS, including *DYSF*, which has been associated with MS severity^4^ (Fig. 3b and Extended Data Fig.4b). When we examined the direction of effects for EBV DNA positivity across autoimmune diseases, we found that *RSBN1*, *SLAMF7*, *CTLA4*, *EOMES*, *CD86*, *TP63*, *TRAF3*, *CLEC16A*, and *IKZF3* showed concordant effects, with increased EBV DNA positivity associated with increased disease risk, while *SP140*, *SH2B3*, and *PTPN11* exhibited discordant directions of effect with less EBV DNA expression (Fig. 2a). The genes highlighted by the GWAS provided biological insights into the regulation of EBV activity. For example, *TERT*, encoding the catalytic subunit of telomerase, emerged as one of the top hits. Indeed, the top variant within this locus, rs7726159, is associated with telomere length^41^ (Supplementary Table 5, Extended Data Fig. 4c). Given that EBV is known to drive telomerase regulation to promote immortalization of infected cells,^42^ this finding may highlight a mechanistic link between EBV replication and telomere biology. We also observed strong support for biologically plausible genes, including *IFI16*, a nuclear DNA sensor that restricts herpesvirus reactivation, and *SP140*, a PML-nuclear body component involved in antiviral chromatin regulation (Extended Data Fig. 4d).^43,45,47^

Stratified LD score regression^44^ revealed that genetic heritability for both EBV DNA positivity and EBV DNA load was concentrated in transcriptionally active genomic regions, particularly at transcription start sites (TSS; enrichment=23.4±4.1 [positivity], 31.3±5.3 [load]), FANTOM5 enhancers (19.9±10.3 [positivity], 32.7±13.8 [load]), and coding regions (17.2±5.2 [positivity], 17.0±6.3 [load]), indicating that genetic variants influencing EBV control tend to localize to regulatory and coding elements with high transcriptional activity (Extended Data Fig. 3B). Pathway enrichment analysis revealed that EBV susceptibility loci were primarily concentrated in immune response–related pathways (Fig. 2c, Extended Data Fig. 4e, Supplementary Tables 7 and 8). Both EBV positivity and EBV DNA load showed significant enrichment for T cell activation, CD8^+^ αβ T cell activation, and T cell proliferation, reflecting a shared involvement of cytotoxic T cell–mediated immune responses. In contrast, cell cycle G1/S phase transition was specifically enriched for EBV positivity, while EBV DNA load additionally involved epithelial-associated pathways such as positive regulation of keratinocyte proliferation and multiciliated epithelial cell differentiation, indicating potential contributions of epithelial cell proliferation and differentiation to viral load regulation.

### Tissue-, Cell-, and Niche-Specific Heritability Analyses

To elucidate the biological contexts underlying host control of EBV activity, we performed partitioned heritability analysis using stratified LD score regression (S-LDSC),^46^ which infers tissue-level enrichment based on external functional annotations. Both EBV positivity and EBV DNA load exhibited marked enrichment in immune-related tissues (Fig. 2d and Extended Data Fig. 4f). For EBV positivity, significant heritability enrichment was observed in spleen, peripheral blood, lymph nodes, synovial fluid, and lung, suggesting that systemic and mucosal immune environments contribute to host susceptibility to EBV activity. In contrast, EBV DNA load showed its strongest enrichment in EBV-transformed lymphocytes, followed by spleen, lymphoid tissue, and blood, indicating a link between viral load regulation and B cell transformation processes.

**Extended Data Figure 5:**
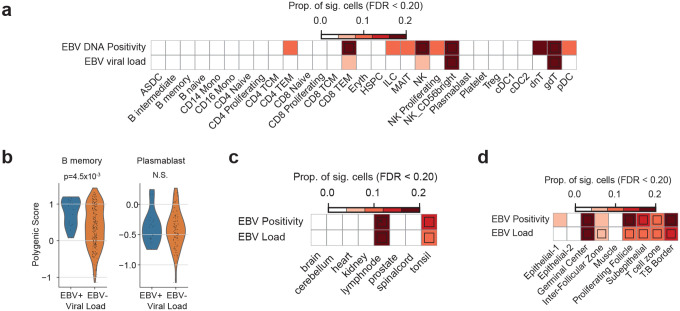
Biological interpretation of GWAS results. (a) Proportion of scDRS-significant cells (*FDR*<0.2) across immune cell types for EBV DNA positivity and viral load. (b) Polygenic scores derived from the EBV load GWAS in B memory and plasmablast populations. The Mann-Whitney U test was applied. (c and d) Enrichment in tissues (c) and anatomical regions in tonsils (d) of EBV polygenic signals estimated by scDRS-spatial. Heatmap colors depict the proportion of significant cells (*FDR*<0.2) evaluated using scDRS. Squares denote significant disease associations (*FDR*<0.05), and cross symbols denote significant heterogeneity in association (*FDR*<0.05).

We next sought to interpret the polygenic signals identified in the EBV GWAS in the context of specific cell types and spatial niches using integrated single-cell and spatial transcriptomic analyses. We first applied single-cell disease relevance score (scDRS)^48^ to single-cell RNA-seq data from PBMCs of EBV-positive and control individuals^49^, analyzing all cell types jointly. This analysis revealed enrichment of EBV-associated polygenic signals in cytotoxic lymphocyte populations, including CD8 effector memory T cells, NK cells, and gd T cells (Extended Data Fig. 5a).

Using the same dataset, we then focused the analysis on B-cell lineages that are directly involved in EBV infection. The dataset included publicly available single-cell RNA-seq profiles from patients with infectious mononucleosis and hemophagocytic lymphohistiocytosis (HLH). Existing pipelines based on Cell Ranger remove overlapping reads, resulting in substantial loss of reads from the compact EBV genome, and they may misalign reads with sequence similarity to human transcripts.^50^ To overcome these limitations, we developed VIRTUS3, an analysis framework optimized for viral detection in single-cell data (https://github.com/yyoshiaki/VIRTUS3). In this workflow, reads are first aligned to the human genome using Cell Ranger. Unmapped reads are then recovered and re-aligned to viral genomes using Alevin. This pipeline can filter out reads with stringent similarity to the human genome, and also manipulate the reads aligned to multiple genes on viral genomes. This approach enabled the detection of 1.84-fold more EBV-positive cells compared with conventional methods (Figs. 3a–c). EBV-positive cells were predominantly detected among plasmablasts, followed by B memory and B intermediate cells, whereas naïve B cells showed almost no EBV positivity (Fig. 3d). Using scDRS, we further evaluated the cell-type–specific distribution of EBV DNA-associated polygenic scores. In memory B cells, polygenic scores were significantly elevated in EBV-positive compared with EBV-negative cells (*p*=3.0×10^−^), while no difference was observed in plasmablasts (Fig. 3e and Extended Data Fig. 5b). These findings suggest that host genetic control of EBV activity is exerted primarily within the memory B-cell compartment rather than at the terminally differentiated plasmablast stage.

We next investigated the immune microenvironments in which host genetic factors regulating EBV activity are concentrated by applying scDRS-spatial, a spatial heritability framework we previously developed^51^. At the tissue level, polygenic signals showed the strongest enrichment in lymph nodes and tonsils, confirming that these lymphoid tissues represent major sites of EBV regulation (Extended Data Fig. 5c). We then analyzed the spatial localization of genetic signals within tonsillar tissue, a key reservoir for EBV infection, latency, and reactivation. Polygenic signals for both EBV DNA positivity and viral load were highly enriched within the germinal center, T:B border, and proliferating follicle regions, which are immune niches characterized by active B cell responses and dynamic B–T cell interactions (Figs. 3f–h and Extended Data Fig. 5d). These findings indicate that host genetic factors controlling EBV activity act predominantly within lymphoid microenvironments supporting T cell–dependent B cell activation. In particular, the germinal center and T:B border emerge as key immunological niches where genetic variation may influence EBV persistence and immune control.

### Antigen presentation by HLA and EBV activity

**Extended Data Figure 6:**
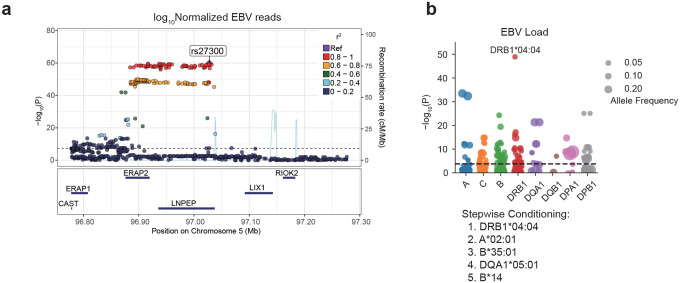
HLA association with EBV DNA load. (a) Locus plot of rs27300 in EBV viral load GWAS. (b) Associations between HLA alleles and EBV DNA load. The size of each point corresponds to the allele frequency, and the top associated alleles are labeled. The alleles highlighted by conditioning were also shown.

**Figure 4:**
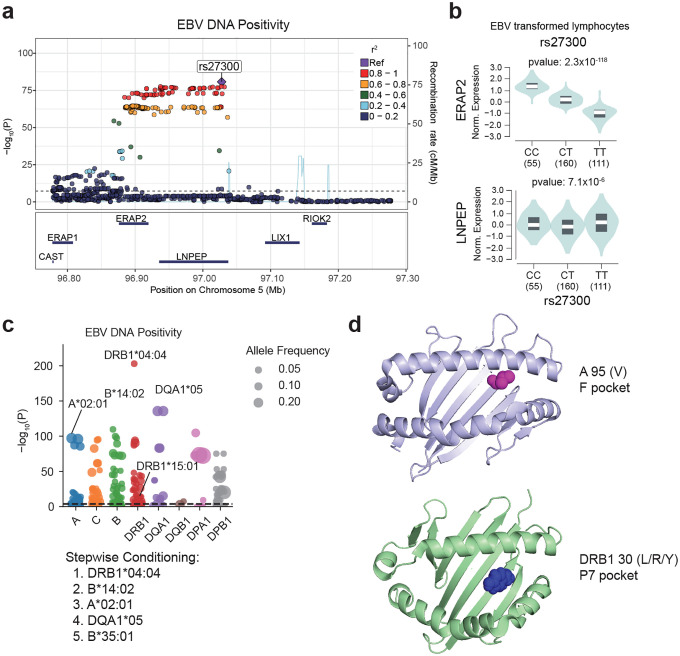
Association of HLA alleles and amino acid variants with EBV DNA positivity and viral load. (a) Locus plot of rs27300 in EBV Positivity GWAS. (b) eQTL plot of rs27300 in *ERAP2* and *LNPEP* in EBV transformed lymphocytes. The eQTL data was downloaded from GTEx Portal.^106^ (c) Associations between HLA alleles and EBV DNA positivity. The size of each point corresponds to the allele frequency, and the top associated alleles are labeled. The alleles highlighted by conditioning were also shown. HLA association analysis using All of Us European participants was performed by imputing classical HLA alleles with SNP2HLA. (d) Amino acid residue associated with EBV DNA positivity/viral load in HLA-A*02:01 (top) and HLA-DRB1*04:04 (bottom). The structure was downloaded from PDB (3MRF for HLA-A*02:01 and 7nzf for HLA-DRB1*04:04).

The strongest associations in both GWASs were located within the HLA locus. In addition, a secondary genome-wide significant signal was identified at rs27300, a variant residing within a linkage disequilibrium block encompassing the *LNPEP* and *ERAP1/2* genes (Fig. 4a and Extended Data Fig. 6a). This variant exhibited a significant eQTL effect on *ERAP2* expression in lymphoblastoid cell lines (LCLs) (*p*=2.33×10^−118^, Fig. 4b), but not with *LNPEP* (*p*=7.07×10^−6^, Fig. 4b). *ERAP2* encodes an aminopeptidase essential for peptide trimming selectively during antigen presentation via MHC class I molecules. This allelic directionality provides additional support for a functional role of *ERAP2* in EBV control. The *ERAP2*-increasing C allele corresponds to the protective direction in the EBV GWAS, suggesting that more efficient peptide trimming enhances antigen presentation and improves the clearance of EBV-infected cells.

**Extended Data Figure 7:**
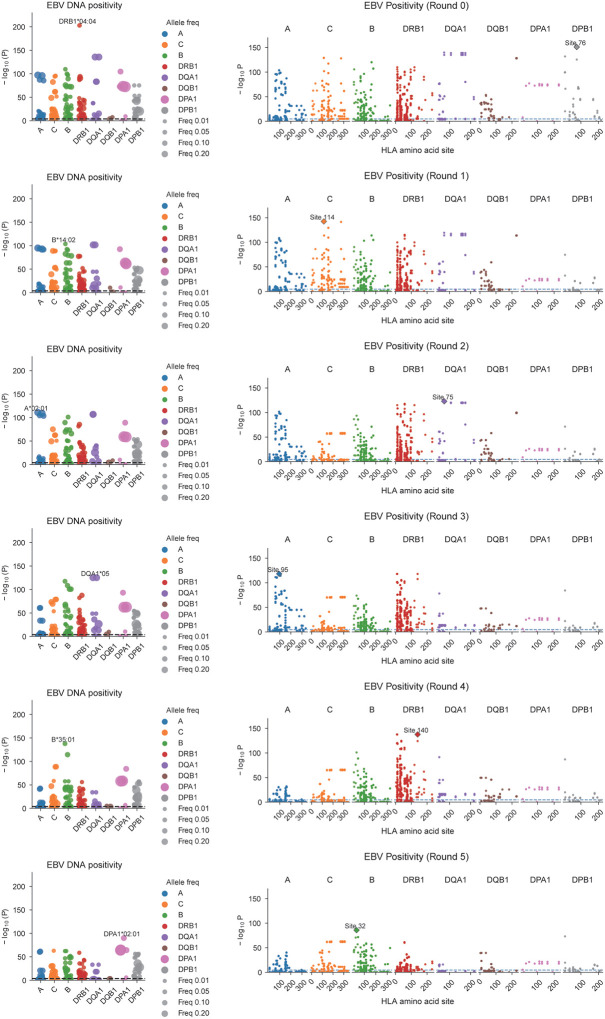
Conditioning of HLA allele and amino acids association with EBV DNA Positivity. Association results for classical HLA alleles across loci (left) and amino acids (right) shown as −log_10_(P values). The most strongly associated alleles in each conditioning round are labeled. HLA amino acid association results for EBV DNA positivity across iterative conditional analyses (Rounds 0–5).

**Extended Data Figure 8:**
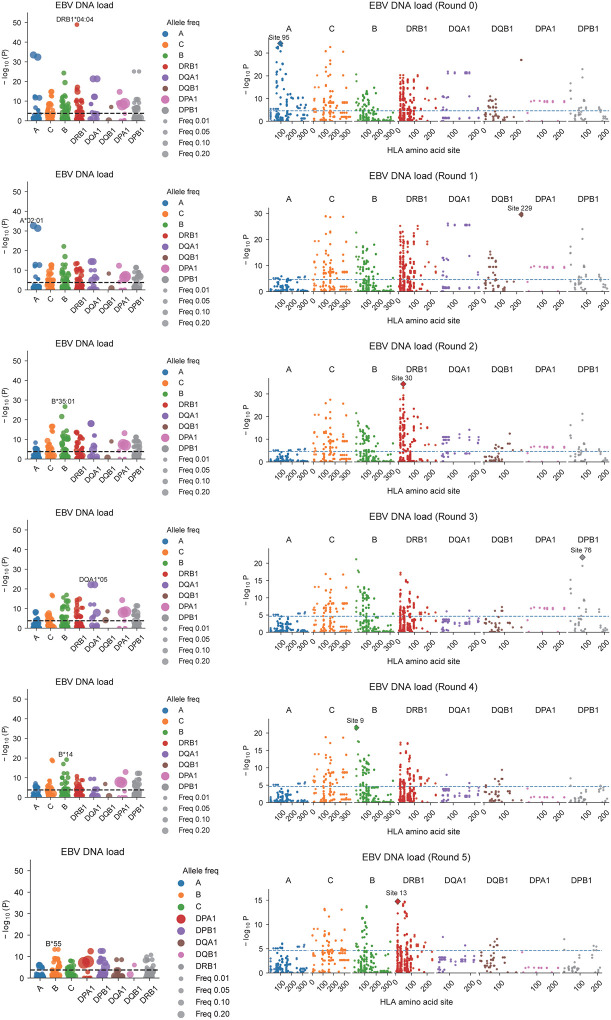
Conditioning of HLA allele and amino acids association with EBV DNA load. Association results for classical HLA alleles across loci (left) and amino acids (right) shown as −log_10_(P values). The most strongly associated alleles in each conditioning round are labeled. HLA amino acid association results for EBV DNA load across iterative conditional analyses (Rounds 0–5).

We also investigated the associations with HLA alleles and amino acid usage and EBV susceptibility. Using All of Us participants of European ancestry, classical HLA alleles were imputed with SNP2HLA,^52^ and their associations with EBV activity (DNA positivity and DNA viral load) were examined. In both traits, HLA-DRB1*04:04 showed the most significant association. After conditioning analysis, the top alleles associated with EBV DNA positivity were HLA-DRB1*04:04, B*14:02, A*02:01, DQA1*05, and B*35:01, while those for EBV DNA load were HLA-DRB1*04:04, A*02:01, B*35:01, DQA1*05:01, and B*14, showing a broadly consistent pattern between the two traits (Fig. 4C, Extended Data Figs. 6B, 7, 8, Supplementary Tables 9, 10). While to a lesser degree, EBV DNA positivity was also seen with the strongest MHC associated allele, DRB1*15:01. Furthermore, an amino acid–level association analysis identified multiple residues significantly correlated with EBV activity (Fig. 4c, Extended Data Figs. 6b, 7, 8, and Supplementary Tables 11, 12). Notably, valine at position 95 of HLA-A*02:01 (A95V), detected in both positivity and viral load analyses, was located within the F pocket of the peptide-binding groove, while position 30 of HLA-DRB1 (L/R/Y variants), detected in the viral load analysis, was located in the P7 pocket (Fig. 4d). Both residues are situated within key structural regions that determine peptide-binding specificity, suggesting that EBV-associated HLA signals may reflect functional variation in peptide-binding pockets influencing viral antigen presentation and immune recognition. These findings suggested that antigen presentation is a key component of EBV regulation and a hotspot of susceptibility.

### Causal inference of EBV activity on autoimmune diseases through Mendelian randomization

**Extended Data Figure 9:**
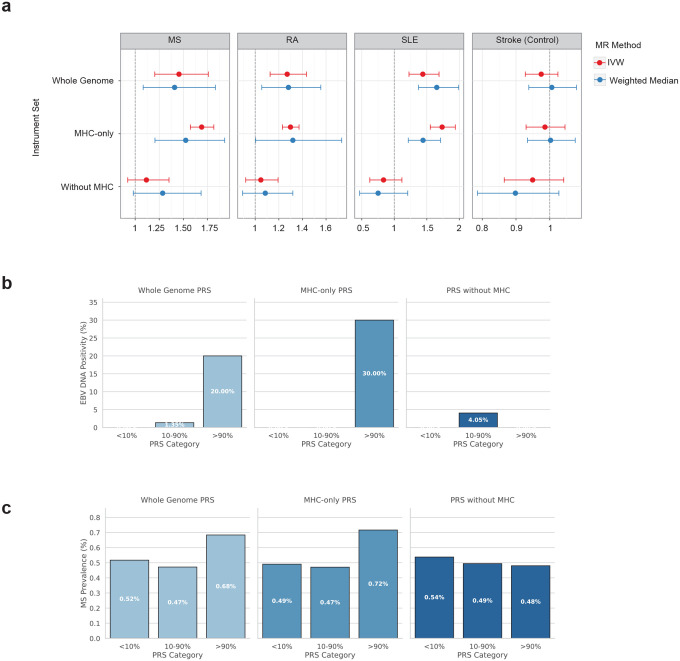
Mendelian randomization and polygenic risk score stratified by HLA region. (a) Forest plots of Mendelian randomization (MR) estimates assessing the causal effect of genetically predicted EBV DNA positivity on autoimmune diseases (multiple sclerosis [MS], rheumatoid arthritis [RA], and systemic lupus erythematosus [SLE]) and stroke (negative control). Three instrument sets were analyzed: whole genome, MHC-only, and genome without the MHC region. Points represent odds ratios; horizontal lines indicate 95% confidence intervals. The dashed vertical line indicates the null effect (OR = 1). (b) EBV DNA positivity measured by qPCR across categories of the EBV DNA positivity PRS in an independent Yale cohort of European-ancestry individuals without MS (N = 94; 3 EBV DNA+ cases). (c) MS prevalence across EBV DNA positivity PRS categories in the UK Biobank European-ancestry cohort (N = 487,181; 2,423 MS cases). For panels b and c, PRSs were constructed using PRScs with the European-ancestry meta-analysis GWAS (b) or All of Us European-ancestry GWAS (c) for three variant sets: whole genome, MHC-only, and non-MHC. Individuals were stratified into bottom 10%, middle 80%, and top 10% of each PRS distribution. Percentages indicate the proportion of EBV DNA-positive individuals (b) or MS cases (c) within each category.

**Figure 5:**
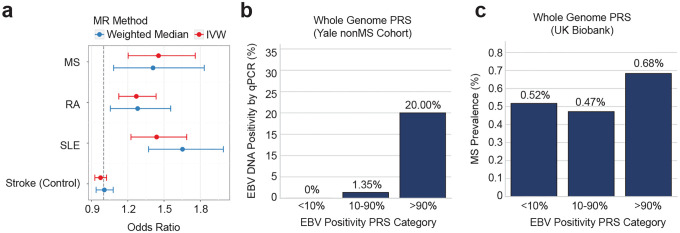
Mendelian randomization and polygenic risk score analyses of EBV DNA positivity. (a) Forest plot of Mendelian randomization (MR) estimates assessing the causal effect of genetically predicted EBV DNA positivity on autoimmune diseases (multiple sclerosis [MS], rheumatoid arthritis [RA], and systemic lupus erythematosus [SLE]) and stroke (negative control). Points represent odds ratios; horizontal lines indicate 95% confidence intervals. The dashed vertical line indicates the null effect (OR = 1). (b) EBV DNA positivity measured by qPCR across categories of the EBV DNA positivity PRS in an independent Yale cohort of European-ancestry individuals without MS (N = 94; 3 EBV DNA+ cases). (c) MS prevalence across EBV DNA positivity PRS categories in the UK Biobank European-ancestry cohort (N = 487,181; 2,423 MS cases). For panels b and c, whole-genome PRSs were constructed using PRScs with the European-ancestry meta-analysis GWAS (b) or All of Us European-ancestry GWAS (c, to avoid sample overlap). Individuals were stratified into bottom 10%, middle 80%, and top 10% of the PRS distribution. Percentages indicate the proportion of EBV DNA-positive individuals (b) or MS cases (c) within each category.

To test whether EBV DNA positivity was causally associated with risk of MS and other major autoimmune diseases, we performed bidirectional Mendelian randomization (MR). For this purpose, we leveraged a set of genetic instruments derived from the European meta-analysis of EBV DNA positivity (27 independent SNPs after accounting for linkage disequilibrium). We observed significant associations between genetically-determined EBV DNA positivity and MS (*OR*=1.45, *95% CI*=1.18–1.80, *p*=5.9×10^−^1), RA (*OR*=1.27, *95% CI*=1.05–1.54, *p*=0.015), and SLE (*OR*=1.44, *95% CI*=1.08–1.91, *p*=0.012) using MR-PRESSO^53^-corrected IVW estimates, with sensitivity analyses using the Weighted Median method confirming the directionality of these associations (MS: *OR*=1.41, *95% CI*=1.08–1.83, *p*=0.011; RA: *OR*=1.28, *95% CI*=1.06–1.56, *p*=0.012; SLE: *OR*=1.65, *95% CI*=1.37–1.99, *p*=1.2×10^−7^) (Fig. 5a). Ischemic stroke was included as a negative control and showed no association with EBV DNA positivity (*OR*=0.97, *95% CI*=0.93–1.02, *p*=0.31). Stratification of instruments showed that robust associations were observed for MHC variants (IVW: MS *OR*=1.69, 95% *CI*=1.57–1.82, *p*=3.5×10^−46^; RA *OR*=1.30, *95% CI*=1.23–1.37, *p*=4.7×10^−21^; SLE *OR*=1.74, *95% CI*=1.55–1.94, *p*=1.9×10^−22^), while non-MHC variants showed no significant causal effects on any autoimmune disease, indicating that the MHC explains a large proportion of the inferred causality for these autoimmune diseases (Extended Data Fig. 9a). In the reverse direction, genetic liability to MS, RA, and SLE was not significantly associated with EBV DNA levels (all *p*>0.05 for IVW and Weighted Median), supporting a unidirectional causal effect of EBV activity on autoimmune disease risk rather than reverse causation.

### Polygenic risk scoring and EBV DNA measured by quantitative PCR (qPCR)

To evaluate whether identified genome-wide significant risk loci for EBV DNA capture biologically meaningful variation in viral load, we assessed polygenic risk scores (PRSs) in an independent cohort with qPCR-based measurement of EBV DNA. We calculated PRSs based on risk variants identified in the meta-analysis of European ancestry using PRScs.^54^ We evaluated association with EBV DNA positivity measured by qPCR in an independent Yale cohort of European ancestry (*N*=94; mean age 40.2±14.7 years; 71% female). For PRS based on 729,766 variants across the whole genome, EBV DNA positivity was 2/10 (20%) in the top decile, 1/74 (1.4%) in the middle group, and 0/10 (0%) in the bottom decile for both positivity and viral load PRS (Fig.5b). Ordinal trend tests remained significant after covariate adjustment (positivity: *p*=0.024; viral load: *p*=0.016). Consistently, a 1 standard deviation increase in the PRS was associated with a higher EBV DNA load after adjustment (log10 EBNA1 copies/mL) (positivity: *β*=0.21, *SE*=0.076, *p*=0.006; load: *β=0.26*, *SE*=0.075, *p*=0.0009). When we stratified variants by their location inside or outside the MHC region, only the PRSs derived from the MHC region showed significant associations with qPCR-confirmed EBV DNA positivity and load (adjusted trend tests: positivity *p*=0.006; load *p*=0.014; effect sizes for 1 SD increase: positivity *β*=0.24, *SE*=0.074, *p*=0.001; load *β*=0.27, *SE*=0.074, *p*=0.0003) (Extended Data Fig. 9b). In contrast, PRSs constructed from variants outside the MHC region showed no significant association (adjusted trend *p*>0.65 for both positivity and load), underscoring the contribution of HLA variation. Taken together, these findings demonstrate that qPCR-measured EBV DNA levels in our independent cohort closely track with the polygenic architecture identified by our EBV DNA GWAS, supporting the validity and biological relevance of the GWAS results.

### Association of PRS of EBV DNA with multiple sclerosis

Next, to investigate how the polygenic signal of EBV activity contributes to MS onset, we analyzed PRSs derived from EBV DNA GWAS. We first constructed PRSs using the EBV DNA GWAS performed in the European ancestry subset of the All of Us cohort and applied them to individuals of European ancestry in the UK Biobank (*N*=487,181; 2,423 MS cases; 484,758 controls). A 1 standard deviation increase in the EBV DNA positivity PRS was associated with a significant increase in MS risk (*OR*=1.11, *95% CI*=1.07–1.16, *p*<0.0001) after covariate adjustments. Stratification of individuals by PRS percentiles (bottom 10%, middle 80%, top 10%) revealed a significant ordinal association between higher EBV DNA PRS and increased risk of MS (Fig. 5c). MS prevalence increased from 0.52% in the bottom decile to 0.68% in the top decile, with individuals in the top decile showing a 33% increased odds of MS compared with those in the bottom decile (*OR*=1.33, *95% CI*=1.13–1.57, *p*=0.0006). When we further separated PRSs into variants inside versus outside the MHC region, the association remained robust for MHC-based scores (*OR per SD*=1.12, *95% CI*=1.07–1.16, *p*<0.0001; top vs bottom decile *OR*=1.44, 95% *CI*=1.22–1.70, *p*<0.0001) but not for scores derived from non-MHC variants (*OR per SD*=0.99, 95% *CI*=0.95–1.03, *p*=0.71), indicating that MHC variation largely accounts for the shared genetic architecture between EBV activity and MS susceptibility. Quintile analyses confirmed this pattern, with individuals in the top quintile of the whole-genome EBV DNA positivity PRS showing 43% higher odds of MS compared with the bottom quintile (*OR*=1.43, *95% CI*=1.26–1.61, *p*<0.0001), an effect entirely attributable to MHC variants (MHC-only Q5 vs Q1: *OR*=1.45, 95% *CI*=1.28–1.64, *p*<0.0001; non-MHC Q5 vs Q1: *OR*=1.01, *95% CI*=0.89–1.14, *p*=0.91). Taken together, these results suggest that the polygenic burden associated with EBV activity meaningfully contributes to MS risk.

### EBV DNA detection in blood from MS patients

We next examined whether EBV DNA levels are directly associated with MS by qPCR. We compared whole-blood EBV DNA positivity between MS patients and non-MS individuals collected at Yale. EBV DNA was detected in 10.9 percent of MS patients (24/220) and 7.65 percent of controls (13/170), showing a higher proportion in MS, although the difference did not reach statistical significance (*p*=0.301, tested by Fisher’s Exact Test).

**Extended Data Figure 10:**
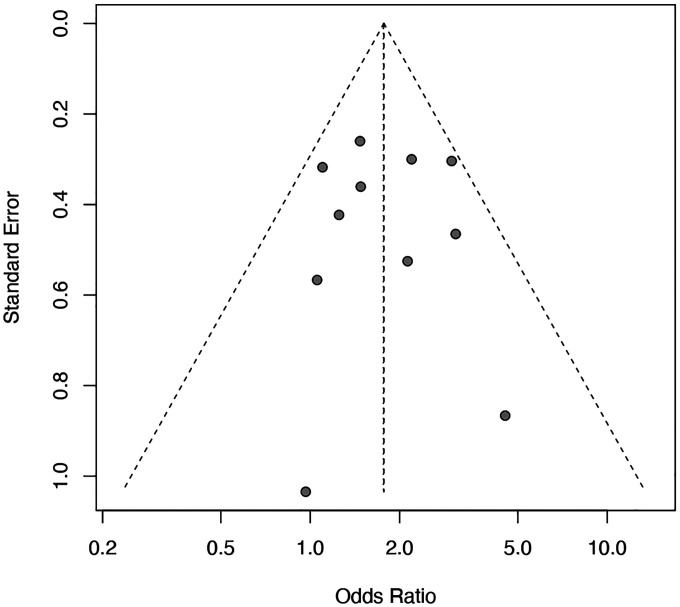
Funnel plot assessing publication bias in studies comparing EBV DNA positivity between MS and controls. Each point represents an individual study included in the meta-analysis. The vertical dashed line indicates the pooled odds ratio under the common-effect model (*OR*=1.77). The dashed diagonal lines denote the 95 percent confidence limits of the expected distribution in the absence of publication bias.

**Figure 6:**
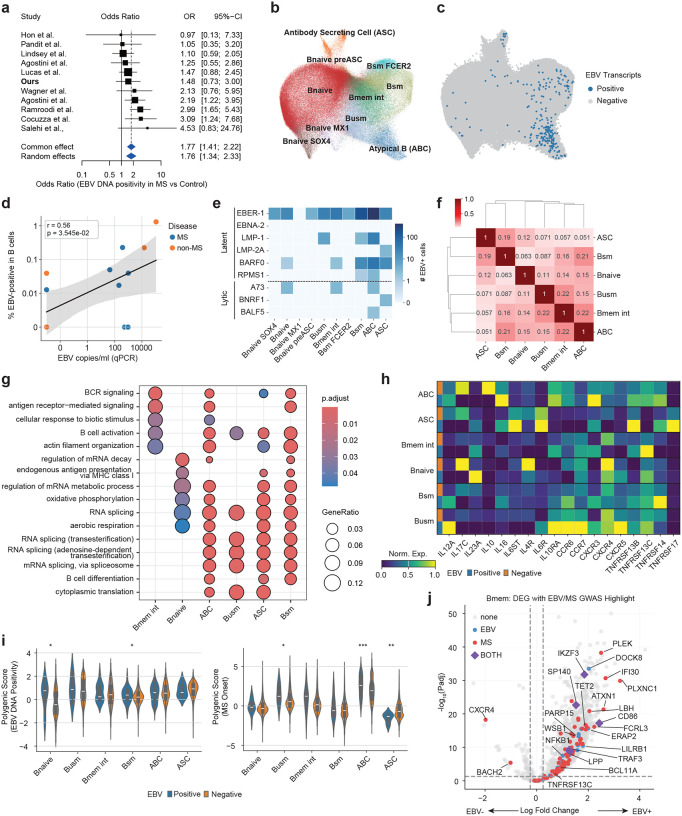
Single-cell EBV detection from people with MS and healthy individuals (a) Forest plot showing OR and 95 percent confidence intervals for EBV DNA positivity comparing patients with multiple sclerosis (MS) and non-MS controls across published studies and our cohort. Squares indicate study-specific ORs with sizes proportional to study weights, and horizontal lines represent 95 percent CIs. The diamonds indicate the pooled effect estimates under fixed-effect and random-effects models. Quantification of heterogeneity: τ^*2*^=0.0169, τ=0.1301, *I*^*2*^=9.8 percent [0.0 percent; 49.4 percent], *H*=1.05 [1.00; 1.41]. A funnel plot is shown in Extended Data Fig. 10. (b and c) UMAP embedding of B-cell subsets from PBMCs (b) and EBV–positive cells (c). (d) Relationship between EBV copies per mL (qPCR) and the percentage of EBV^+^ B cells, stratified by disease status (MS vs non-MS). The shaded region denotes a 95 percent confidence interval. Pearson’s *r* and *p-value* are shown in the figure. (e) Heatmap of viral transcript detection across B-cell subsets, displaying number of cells expressing canonical EBV latent and lytic transcripts. (f) Pearson’s correlation of differentially expressed genes in EBV–positive cells compared to EBV–negative cells across the B cell population. (g) Gene Ontology (GO) enrichment analysis comparing EBV–positive versus EBV–negative cells for each B cell population. Dot size represents gene ratio, and color indicates adjusted *p*-value. (h) Expression heatmap of cytokines, chemokine receptors, and immunomodulatory molecules differentially expressed in EBV–positive cells. Expression was normalized for each gene. (i) scDRS-based polygenic score enrichment for EBV DNA positivity (left) and MS onset genes (right) across B-cell subsets, comparing EBV–positive and EBV–negative cells. Statistical test was performed by the Mann-Whitney U test. (j) Volcano plot of differentially expressed genes in EBV–positive versus EBV–negative memory B cells (Busm, Bmem int, Bsm, Bsm *FCER2*, and ABC are pooled). Genes highlighted in the EBV and MS GWAS are annotated.

To more rigorously assess this association, we conducted a meta-analysis of 11 studies encompassing 1,725 individuals and 526 EBV DNA–positive events (Fig. 6a, Extended Data Fig. 10, Table S13). The fixed-effect model yielded an odds ratio of 1.77 (95% *CI*=1.41–2.22, *p*<0.0001), and the random-effects model produced a nearly identical estimate (*OR*=1.76, 95 % *CI*=1.34–2.33, *p*=0.0010). Heterogeneity was low (*I*^*2*^ = 9.8 percent), indicating consistent effects across studies. Although the effect size in MS is more modest than in other autoimmune diseases such as SLE or RA, where odds ratios typically range from 3.5 to 5,^26,55^ well-controlled case–control comparisons consistently demonstrate higher circulating EBV DNA in MS. This pattern reinforces the concept that EBV activity is elevated around the time of MS onset.

### Single-cell resolution cellular dynamics in EBV-infected B cells in people with MS

**Extended Data Figure 11:**
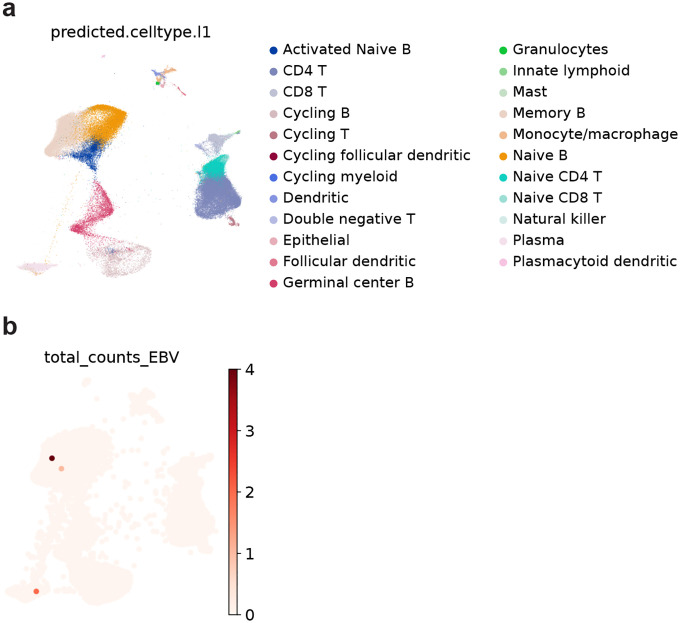
EBV profiling of tonsils from MS and nonMS individuals (a and b) UMAP plot showing predicted clusters (a) and detected EBV–positive cells (b).

We next sought to directly characterize the interaction between EBV infection and MS-related genetic programs by performing single-cell analyses of PBMCs from MS and healthy individuals with EBV detection at single-cell resolution. A comprehensive survey of public scRNA-seq datasets revealed virtually no EBV-positive cells.^56^ This was unlikely to reflect a true biological absence but rather a technical limitation of conventional single-cell RNA-seq, which captures only polyadenylated transcripts and therefore fails to detect the major latent EBV noncoding RNA *EBER1*, which lacks a polyA tail. To overcome this limitation, we introduced a spike-in probe enabling specific capture of *EBER1*, which is a similar approach to EBV-seq.^57^ Yet even with this improved method, EBV-infected cells remained exceedingly rare, with none detected in PBMCs (0 of 51,950 cells from 3 donors) and only three cells detected in tonsils (3 of 134,073 cells from 12 donors) from both healthy donors and MS patients, underscoring a fundamental limitation of single-cell approaches when the infected population is extremely sparse (Extended Data Fig. 11).

**Extended Data Figure 12:**
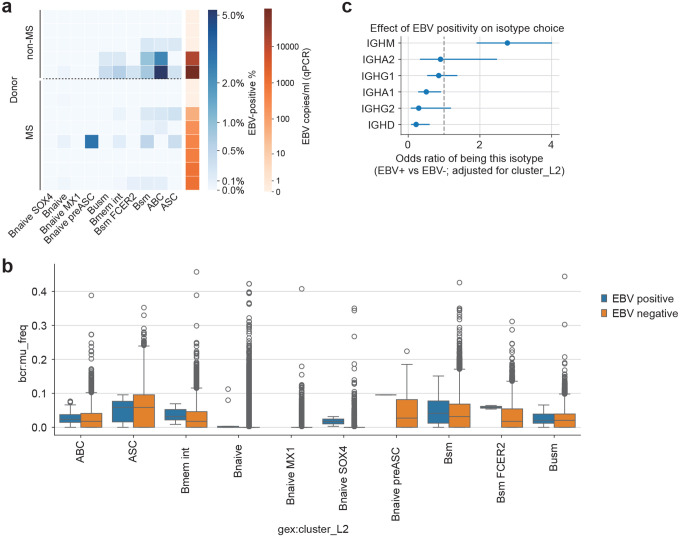
EBV profiling of blood B cells from MS and nonMS individuals (a) Percentage of EBV–positive cells across B cell populations per samples. Rows indicate each donor. The heatmap in orange shows qPCR based copy number of EBV DNA in blood. (b) Forest plot showing the association of isotype with EBV DNA positivity. Clusters were treated as a covariete to extract global isotype preference. IGHE, IGHG3, IGHG4 were removed from the plot because no EBV–positive cells were detected. (c) Somatic hyper mutation frequency distribution across clusters between EBV–positive and –negative cells.

Previous studies have shown that blood EBV DNA load strongly correlates with the number of *EBER1*-positive cells,^58^ suggesting that EBV DNA–positive samples may enable the recovery of EBV-infected cells at single-cell resolution. We therefore analyzed banked frozen PBMCs from EBV–positive individuals screened by qPCR analysis. We performed single-cell RNAseq with the *EBER1* spike-in probe, prioritizing EBV DNA–positive MS and non-MS samples. With this technique, we could detect 247 DNA-positive cells from 201,398 B cells (0.12%) (Figs. 6b and c). The EBV–positive cells were predominantly memory B cells and plasma cells (Fig. 6c and Extended Data Fig. 12a), and the number of EBV–positive cells were positively correlated with qPCR-based EBV DNA load (*r*=0.56, *p*=0.035, Fig. 6d). While we did not find a significant difference in terms of somatic hyper mutation (*p*=0.0879, Extended Data Fig. 12b), *IGHM* were more frequent and *IGHA1*, and *IGHD* were less frequent (Extended Data Fig. 12c). Most of the EBV–positive cells expressed latent EBV genes including *EBER1*, while some atypical B cells, naïve B cells, intermediate memory B cells, and antibody-secreting B cells expressed lytic genes (Fig. 6e).

Next, we examined how EBV-positive B cells alter their transcriptional programs. EBV infection induced two broad patterns of gene expression changes, separating mature populations including antibody-secreting cells, switched memory B cells from more immature populations naïve (naïve, unswitched-memory, intermediate memory, and atypical B cells (Fig. 6f, Table S15). Pathway analysis revealed marked upregulation of BCR signaling, antigen receptor–mediated signaling, B-cell activation, and RNA splicing in memory B cells and antibody-secreting cells, whereas antigen presentation via MHC class I was particularly enriched in naive B cells (Fig. 6g, Table S16). A focused analysis of cytokines and receptors involved in immune interactions (Fig. 6h) showed increased expression of *IL12A* and *IL23A*, in EBV infected B cells which promote Th1 and Th17 differentiation, together with reduced expression of the immunoregulatory cytokine *IL10* and its receptor *IL10RA*. EBV-positive cells also upregulated *IL16*, *IL6ST* (gp130), and *IL6R*, molecules implicated in MS pathogenesis. While *CXCR4* was downregulated, molecules involved in recruiting and activating pathogenic T-cell subsets, including *CCR6* (Th17-associated) and *CXCR3* (Th1-associated), were also increased. In addition, EBV-positive cells showed elevated expression of receptors crucial for B-cell differentiation and survival, including *CXCR5*, *TNFRSF13B* (TACI), *TNFRSF13C* (BAFF-R), *TNFRSF14* (HVEM), and *TNFRSF17* (BCMA).

Finally, we investigated how host genetic factors interact with EBV infection across B-cell subsets. Using scDRS to evaluate EBV GWAS signals, we found that the EBV DNA positivity–associated polygenic signal was significantly enriched in EBV-positive B naïve and switched memory B cells, whereas no meaningful enrichment was observed in antibody-secreting cells (Fig. 6i). These findings suggest that early infection responses in naïve B cells and persistent infection in switched memory B cells represent key cellular contexts in which EBV regulation is exerted. We then performed a similar analysis using MS onset–associated gene sets. Polygenic scores for MS were markedly elevated in EBV-positive unswitched memory B cells and switched memory B cells, while antibody-secreting cells showed a reduction in MS-related signal (Fig. 6i). Specifically, MS risk gene expression was increased specifically within EBV-infected cells in atypical B cells and unswitched-memory B cells subsets, indicating that EBV-infected B cells constitute a biological context where MS risk genes are preferentially engaged. Notably, many MS susceptibility genes, EBV susceptibility genes, and shared loci between the two GWAS, including *IKZF3*, *SP140*, *CD86*, *NFKB1*, *TRAF3*, and *LPP*, showed increased expression in EBV-positive cells (Fig. 6j). These observations indicate that EBV infection activates a gene network that intersects with MS susceptibility specifically within defined B-cell subsets. Taken together, these results demonstrate that EBV infection enhances both antiviral polygenic signals and MS susceptibility gene expression in select B-cell subsets, revealing a cellular setting in which viral regulation and autoimmune genetic risk converge.

## Discussion

Here, we developed a pipeline to quantify EBV DNA from whole-genome sequencing data and applied it to population-scale cohorts. A cross-ancestry GWAS of EBV DNA positivity in 617,186 individuals identified 39 susceptibility risk loci. We validated this finding in our independent cohort and found that qPCR-confirmed EBV DNA-positive individuals were markedly enriched in the top decile of EBV polygenic risk scores containing these newly discovered loci. Surprisingly, there was a significant overlap with genetic variants associated with MS, RA, and SLE susceptibility alleles, and Mendelian randomization analyses further supported a causal influence of EBV activity on MS, largely driven by HLA variation. By establishing a single-cell RNA-seq method optimized for *EBER1* detection, we found that EBV DNA expression was primarily in memory, atypical, and antibody-secreting B cells and that EBV-infected memory B cells strongly upregulated cytokines and risk genes from both the EBV and MS GWAS. These data demonstrate that EBV–infected B cells are a key modulator of T cells and that MS susceptibility genes are preferentially activated within EBV-infected B cells, defining a genetic and cellular framework linking EBV infection to MS and potentially other autoimmune diseases.

MS is the autoimmune disease for which EBV is most strongly implicated as a necessary environmental factor, and both tissue and cell specificity of EBV regulation further align with MS-specific immunobiology in which memory B cells drive CNS infiltration.^59^ Moreover, it has been clearly demonstrated that the EBV virus harbors sequences that are cross-reactive with myelin epitopes including GlialCAM that are likely also playing a critical role in the pathogensis of MS^18,19^. Thus, EBV uniquely may drive activation of myelin reactive T cells both by breaking immune tolerance and by presenting crossreactive antigens.

Although EBV DNA showed no positive association with MS in our PheWAS,^60^ well-controlled case-control meta-analyses have consistently reported elevated EBV DNA levels in MS patients. This discrepancy is likely explained by the time course of EBV involvement in the onset of MS. Specifically, EBV seroconversion occurs an average of 7.5 years before clinical onset,^12^ MS diagnosis requires CNS lesions disseminated in time and space,^61^ and a prodromal period precedes diagnosis.^62^ As a result, EBV load at the time of diagnosis is likely to be partially deflated. Furthermore, individuals with MS in cohorts such as All of Us include patients receiving long-term immunotherapy and those many years after diagnosis, making it difficult to capture EBV activity during the true initiation phase. These considerations highlight the importance of examining newly diagnosed, treatment-naïve patients or MS high-risk individuals prior to disease onset to accurately evaluate EBV activity in early MS.

Our large-scale WGS analysis also clarified the broader epidemiology and biology of EBV infection across populations. In the United States, individuals of African, Latino/admixed American, and Asian ancestry showed higher EBV positivity rates, consistent with serology-based findings.^63^ Female individuals showed lower EBV DNA positivity despite a higher prevalence of autoimmunity. This pattern suggests that sex differences in EBV susceptibility and the host immune response to EBV may shape autoimmune risk. We also found that a small subset of “super-carriers” accounted for a disproportionate fraction of EBV DNA detected in the population.

Cross-ancestry GWAS clarified the host genetic architecture underlying EBV activity. Strong enrichment of associations in the HLA region, in T-cell–related pathways including association in *CTLA4*, *EOMES*, and *CD86*, and at the T–B border of secondary lymphoid tissues, underscores the central role of B cells in antigen presentation to T cells in EBV regulation. EBV employs multiple immune-evasion strategies, including BNLF2a-mediated inhibition of TAP1.^64^ Here, we showed that HLA polymorphisms shape antigen presentation efficiency and directly influence EBV activity. HLA-DRB1*04:04, a known risk allele for RA and MS,^65,66^ and an allele that presents myelin-specific TCRs in MS patients,^67^ was associated with higher EBV activity. The risk allele HLA-DRB1*15:01 was also associated with higher EBV presentation, though to a lesser extent than the HLA-DRB1*04:04 allele, suggesting other factors contribute to the genetic risk associated with these MHC haplotypes. In contrast, HLA-A*02:01 was associated with reduced EBV activity and is protective against MS^68^ and EBV^+^ Hodgkin lymphoma.^69^ HLA molecules shape antigen-presentation preferences and thymic TCR repertoire formation.^70^ In addition, components of the antigen-processing pathway, including *ERAP2*, emerged as important regulators of EBV control. Our findings indicate that HLA diversity and related genes exert strong effects on EBV susceptibility and that several EBV-associated alleles overlap with autoimmune risk alleles, confirming suggested shared genetic pathways linking EBV control and autoimmunity.^71^ Moreover, PRS and MR analysis highlighted the HLA contribution to autoimmune diseases including MS.

Non-HLA loci further underscore that EBV regulation relies heavily on intrinsic programs of the host B cell. Autoimmune diseases and infections exhibit broad sharing of genetic risk variants across conditions.^72^ Genes in which the direction of effect was concordant between EBV DNA positivity and autoimmune disease risk, including *RSBN1*, *SLAMF7*, *CTLA4*, *EOMES*, *CD86*, *TP63*, *TRAF3*, *CLEC16A*, and *IKZF3*, converged on pathways governing B-cell differentiation, germinal-center dynamics,^73^ NF-κB signaling,^74^ T cell effector function, and antigen presentation. This pattern suggests that B-cell differentiation and activation programs provide a shared biological foundation that simultaneously promotes EBV persistence and increases autoimmune susceptibility. In contrast, genes showing discordant effects, such as *SP140*, *SH2B3* and *PTPN11*, map to pathways involved in immunoregulatory thresholds and negative feedback, raising the possibility that trade-offs between antiviral responsiveness and autoimmune risk underlie their opposing directions of effect. *SP140* localizes to promyelocytic leukemia protein nuclear bodies, a key site of viral genome surveillance, and can repress viral gene activation.^75,76^ There were also loci whose directional relationship with autoimmunity was unclear but nonetheless pointed to plausible mechanisms of EBV regulation. For example, *IFI16* functions as an intrinsic viral DNA sensor that can detect EBV genomes in the nucleus, restrict lytic reactivation, and participate in epigenetic silencing of viral DNA.^77^ Associations in the *TERT* locus further suggests that variation in telomere biology may influence EBV replication and the long-term survival of infected B cells, consistent with the ability of EBV to co-opt telomerase activity during immortalization. Together, these loci highlight a second layer of EBV control, the intrinsic programs of the infected B cell, which operate in parallel with HLA-mediated antigen presentation. This integrated view suggests that host genetic variation shapes not only antiviral immunity but also the cellular context that EBV exploits to sustain lifelong infection and influence on autoimmunity.

Building on these findings, we further leveraged three key advances, improved single-cell detection of viral transcripts, an optimized bioinformatics pipeline, and sample prioritization based on EBV DNA by qPCR, to achieve the direct identification of EBV-infected cells in patients with MS at single-cell resolution. By analyzing a large cohort enriched for untreated, newly diagnosed MS cases, we were able to capture EBV-positive cells during the clinically relevant early phase of disease. These infected cells were concentrated within memory B cells, ABCs and ASCs.

EBV-infected B cells showed a coordinated activation of immune communication pathways, including upregulation of *IL12A* and *IL23A*, which drive Th1 and Th17 differentiation. IL-12 has been shown to diminish Treg suppressive capacity in inflammatory settings, shifting the balance toward effector responses.^78^ This pro-inflammatory shift was further reinforced by reduced expression of *IL10* and its receptor *IL10R*, consistent with impaired regulatory B-cell function described in MS.^79^ EBV-positive cells also increased *IL16*, *IL6ST* (gp130), and IL6R, molecules implicated in MS pathogenesis, and upregulated the chemokine receptors *CCR6* and *CXCR3*, which position B cells within Th17- and Th1-rich inflammatory niches rather than homeostatic environments. In parallel, EBV-positive B cells showed elevated expression of receptors crucial for B-cell differentiation and survival, including *CXCR5*, *TNFRSF13B* (TACI), *TNFRSF13C* (BAFF-R), *TNFRSF14* (HVEM), and *TNFRSF17* (BCMA). Together, these findings indicate that EBV infection simultaneously activates programs that potentiate pathogenic T-cell–B-cell interactions, promote trafficking to inflamed sites, and reinforce intrinsic B-cell maturation and survival, revealing a broad rewiring of B-cell immunobiology relevant to MS.

Notably, both MS susceptibility genes and EBV activity–associated genes were highly expressed within these EBV-infected memory B cells. This represents the first direct evidence that host genetic risk for MS and genetic determinants of EBV activity converge within EBV-infected memory B cells. Given that memory B cells serve as potent, antigen-specific professional antigen-presenting cells, targeting this compartment provides a compelling biological rationale for the remarkable efficacy of B-cell–depleting therapies in MS.

Taken together, our work identifies the epidemiology of EBV across ancestries and elucidates the genetic basis of EBV B cell expression. By integrating multiple omics data, we mapped the key immunological “hotspots” where this regulation occurs. These findings support the existence of shared biological pathways linking EBV to autoimmune diseases including MS. As the genetic variants associated with MS are linked to EBV DNA expression, our results are also consistent with the higher EBV DNA expression in MS B cells. Future studies using Massive Parallel Reporter Assays of autoimmune genetic variants will allow the determination of how EBV gene products interact with disease associated haplotypes in driving B cells to a state of activated, antigen presentation state potentially breaking immune tolerance triggering autoreactive T cells.

## Methods

### Human Subjects

In this study, we analyzed whole-genome sequencing (WGS) data and linked electronic health record information from participants enrolled in the All of Us Research Program and the UK Biobank. All analyses were conducted in accordance with each data resource’s governance framework (All of Us: Registered Tier; UK Biobank: Application 58743), and only authorized researchers accessed the data within secure computing environments. For All of Us, WGS data derived from both blood and saliva were used, whereas UK Biobank provided WGS data derived from blood DNA. In both cohorts, all datasets were de-identified, and no personally identifiable information was available to researchers. Clinical information included ICD-9/10 diagnosis codes, age, sex, self-reported ancestry, and laboratory measurements, limited to variables approved for research use within each biobank.

A cohort of individuals at increased risk for MS was recruited at Yale University. This cohort study consists of (1) family members of MS patients and (2) asymptomatic individuals incidentally discovered to have morphologically suspicious lesions on brain MRI. Participants donate biospecimens and are prospectively followed. European-ancestry cohort members were selected for PRS analysis. A cohort of patients with MS used for qPCR and scRNAseq was also recruited at Yale School of Medicine.

### EBV DNA quantification from WGS

The human reference genome, GRCh38, which is used in AoU and UKB, contains the EBV decoy sequence, named chrEBV. To detect EBV reads in WGS samples, we investigated reads mapped to chrEBV stored in the CRAM files. A WGS sample can sometimes contain very abundant EBV reads due to viremia and have the potential to cause a heavy computational burden. To decrease the computational cost, samples with more than 350,000 reads mapped to chrEBV were randomly downsampled using samtools to 350,000 reads. Reads were then filtered to retain only proper pairs, passing standard quality control metrics, with duplicates, secondary, and supplementary alignments removed. Mapping length was calculated from the CIGAR string, and only reads with an aligned length of ≥145 bp were retained. The EBV genome carries a repetitive region, named BamHI W repeat (positions 12,000–35,355 bp). EBV reads derived from this region can be multi-mapping due to the repetitive nature of the BamHI W repeat region in the EBV reference genome. Our pipeline accounts for this repetitive nature and avoids counting such multi-mapping reads repeatedly. Finally, paired-end reads passing all filters were defined as EBV reads, and counts were summarized separately inside and outside the BamHI W repeat. In the case of analysis with downsampling, it estimates the number of EBV reads in the original CRAM file by accounting for the downsampling ratio.

### Association analysis of EBV DNA

We analyzed the association of EBV positivity with age_at_collection, genetically predicted ancestry, which is provided by All of US, and sex. Observations with missing values were excluded. Logistic regression (generalized linear model with binomial distribution and logit link) was fitted separately for blood samples and saliva samples. The model was specified as:

$$\text{logit}\{P({\text{EBV}}_{\text{pos}}=1)\}=\beta_{0}+\beta_{1}\cdot \text{age\_scaled}+\beta_{2}\cdot \text{ancestry\_pred}+\beta_{3}\cdot \text{sex}$$

To facilitate interpretation, age was scaled in 10-year units (age_scaled = age_at_collection / 10). European ancestry (eur) and male sex were used as reference categories. Robust (HC3) standard errors were applied. Coefficients were reported as odds ratios (ORs) with 95% confidence intervals. Analyses were conducted in Python using the package statsmodels. Logistic regression models were estimated using statsmodels GLM (Binomial, logit) with robust HC3 standard errors.

### Generalized Pareto distribution (GPD) analysis

To assess the tail behavior of EBV DNA distributions, we applied a peaks-over-threshold (POT) approach using the Generalized Pareto distribution (GPD). Normalized EBV DNA reads (normalized by the number of human-genome coverage) were used. For each sample type (blood, saliva), the 95th percentile of the distribution was chosen as the threshold u, and exceedances above u were modeled with the GPD parameterized by shape (ξ) and scale (β). Parameter estimation was performed by maximum likelihood, and 95% confidence intervals of ξ were obtained via profile likelihood. For conceptual illustration, simulated GPD curves with ξ = −0.3, 0, 1.0, and a common scale were generated to highlight the difference between finite, exponential, and heavy-tailed distributions.

### Genotype Data Processing and Quality Control

Genotype data were processed independently for the All of Us Research Program^34^ and the UK Biobank,^35^ following harmonized quality control procedures to enable cross-cohort analyses. Unless otherwise noted, equivalent filters and thresholds were applied across both datasets. The variant set for downstream analyses was defined based on the All of Us ACAF PGEN callset and applied consistently to both cohorts.

For the analysis of All of Us, we utilized the All of Us ACAF PGEN callset, which includes variants with “AF>1% or AC>100 in any ancestry,” filtered using the FT flag. Participants were excluded if DNA was derived from saliva, retaining only blood-derived samples. Following previous studies, we removed individuals with immunodeficiency disorders (ICD-10 codes D80–D89) or those with ‘ID_057.1’ (Human immunodeficiency virus infection) in PhecodeX. Related and flagged individuals were excluded using the All of Us auxiliary files (relatedness_flagged_samples.tsv and flagged_samples.tsv). We retained only samples with inferred sex chromosomes XX or XY, sex at birth recorded as male or female, and available age-at-collection information.

Variant-level quality control was performed per ancestry with the following parameters: MAF between 0.01 and 0.99, Hardy–Weinberg equilibrium (HWE) p>1×10^−^ (using keep-fewhet), biallelic variants only, and a genotype missingness rate <5%. An ancestry-specific variant list was generated for each group, and the union across all ancestries and cohorts was used for subsequent analyses.

For the UK Biobank cohort, EBV DNA was quantified from WGS data using the same pipeline as All of Us, counting reads mapping to the EBV reference genome (chrEBV). EBV read counts were normalized by mean sequencing coverage and log10-transformed (log10[normalized reads + 1]) for the quantitative trait analysis; a binary EBV DNA positivity variable was defined as having at least one EBV-mapped read. Sample-level quality control included: retention of individuals of European genetic ancestry based on the UK Biobank genetic ethnicity field (Data-Field 22006); exclusion of one member from each pair of related individuals (*kinship coefficient*≥0.0442) using the UK Biobank relatedness file; exclusion of individuals with sex chromosome aneuploidy or discordance between reported and genetic sex; removal of WGS preparation plates with unusually high EBV positivity rates (>28%); and exclusion of individuals in the top 1% of normalized EBV reads. As in All of Us, we excluded individuals with immunodeficiency disorders (ICD-10 codes D80–D89) or HIV infection (ICD-10 codes B20–B24), as well as those taking immunosuppressant medications at baseline (Data-Field 20003). Principal components were computed on unrelated European individuals and projected onto all European samples. For GWAS, we used imputed genotype data from UK Biobank. For REGENIE Step 1, we used directly genotyped variants after quality control (*MAF*≥0.05, *genotype missingness*<5%, *MAC*≥40, HWE *p*>1×10^−2^, biallelic SNPs only) and LD pruning (window 200 kb, step 50 variants, *r*^*2*^<0.2), yielding 346,104 variants. For REGENIE Step 2 association testing, we applied filters of MAC ≥ 40 and imputation INFO ≥ 0.8. For the meta-analysis, we restricted to the same set of variants defined by the All of Us ACAF callset by matching on chromosome, position, and alleles.

### Genome-Wide Association Analysis and meta-analysis

Genome-wide association analyses were performed using REGENIE^37^ with comparable parameters across cohorts/population. In step 1, LD-pruned variants were selected using ancestry-specific thresholds.^80^ For individuals of African ancestry, we used --indep-pairwise 1000 100 0.15; for non-African ancestries, --indep-pairwise 1000 100 0.3. Covariates included the first 20 principal components, sex at birth, and age at collection (including quadratic and age×sex interaction terms), with additional cohort-specific covariates such as sequencing batch metrics, fasting time, lymphocyte percentage, smoking status, time of blood draw, and month of collection to account for technical and seasonal variation. Step 2 analyses were restricted to SNPs that passed QC in at least four of the six ancestry groups. For the cross ancestry meta analysis, cohort/population with the participants more than 10,000 (European from UK Biobank and European, African, and Latino/Admixed American from All of Us) were used. We performed the meta analysis using MR-MEGA, which accounts for ancestry heterogeneity through genetic principal components.^39^ For the meta-analysis restricted to Europeans (UK Biobank + All of Us), METAL^38^ was used with an inverse-variance weighted fixed-effects model. The visualization and the selection of lead SNPs were performed using gwaslab.^81^ The annotation of lead SNPs was performed with VEP,^82^ OpenTable^83^ and ImmuNexUT platform.^84^

### LD Score Regression and Tissue-specific Heritability Analyses

We performed LDSC analyses to estimate SNP-based heritability and partition it across tissue- and =cell-type–specific annotations.^40,44,46^ GWAS summary statistics were derived from a meta-analysis of the All of Us and UK Biobank European ancestry cohorts, combined using METAL as described above. To ensure consistency, only HapMap3 (HM3) variants were retained during harmonization. Reference LD scores and regression weights for European populations were obtained from the Broad Institute’s public Google Cloud repository (https://console.cloud.google.com/storage/browser/broad-alkesgroup-public-requester-pays). Baseline model v1.2 annotations were used to account for known genomic features.^44^ For each trait, we estimated SNP heritability and genetic correlations using LDSC. Tissue- and cell-type–specific heritability enrichment was assessed using LDSC (--h2-cts) with Multi_tissue_gene_expr annotations.^46^

### Pathway analysis

First, we performed SNP annotation with gene locations (NCBI37.3, https://ctg.cncr.nl/software/MAGMA/aux_files/NCBI37.3.zip) and the reference data created from 1000 genomics Phase3 (g1000_eur, https://ctg.cncr.nl/software/MAGMA/ref_data/g1000_eur.zip) using magma --annotate (with the option, window=10,10).^85^ For the pathway-level analyses, we used MAGMA v1.10 on the same European-ancestry METAL meta-analysis summary statistics used for LDSC. SNPs were first annotated to genes using NCBI37.3 gene locations with a ± 10 kb window and the 1000 Genomes Phase 3 European reference panel (g1000_eur) as the linkage disequilibrium (LD) reference, and variants within the extended MHC region (chr 6: 25–34 Mb) were excluded. Gene-based association tests were then performed with MAGMA using SNP-level p values and sample sizes while accounting for LD from g1000_eur, yielding gene-level z statistics. These results were subsequently used for competitive gene-set analyses that were carried out using MSigDB v2025.1 gene sets, including Hallmark, KEGG canonical pathways, and Gene Ontology (biological process, molecular function, and cellular component) collections.

### PheWAS

We conducted analyses in individuals of European ancestry from the All of Us cohort. ICD-9 and ICD-10 codes were harmonized using PhecodeX.^86^ The R package SPAtest was used to perform score tests based on the saddlepoint approximation, and beta coefficients were estimated using Firth’s method. Visualization was performed with matplotlib.

### EBV detection from scRNAseq data.

We developed a pipeline, VIRTUS3, to detect EBV transcripts from 10x Genomics scRNA-seq data. First, we performed alignment to the human reference (GRCh38, refdata-gex-GRCh38–2020-A) and cell barcode calling using Cell Ranger count. The resulting barcodes were used as a whitelist for viral quantification. Unmapped reads were extracted from the position-sorted BAM file with samtools view -f 4 and converted back to FASTQ with cellranger bamtofastq. These unmapped reads were then quantified against an EBV reference (NC_007605.1, B95–8 strain, CDS plus EBER1/2) using Salmon/Alevin^87^ with the barcode whitelist. The pipeline was applied to the 10x 3’ data of PBMC from EBV-HLH, EBV-IM, and healthy individuals.^49^ We compared the number of EBV-positive cells by VIRTUS3 with the reported number in Suzuki *et al*.^49^ In their analysis, EBV detection was performed using Cell Ranger v5.0 with the custom reference made by the human genome (GRCh38) and the genome of the EBV representative strain (NC_007605).

### Polygenic signal enrichment analysis using scDRS and scDRS-spatial

The polygenic signals were calculated using scDRS^48^ and scDRS-spatial.^51^ The gene scores derived from MAGMA were used. For PBMC scRNAseq data and the visualization of spatial transcriptome data, we used imputed gene expression calculated by MAGIC.^88^ For tissue- and region-specific analysis, we used raw counts for scDRS. The validation of the usage of MAGIC is described in our previous publication.^51^ Polygenic enrichment for each cell was calculated using scDRS (v1.0.3) with --n-ctrl 1000. We used the number of detected genes per cell as a covariate. Group-level statistics were summarized using scdrs perform-downstream and visualized with scdrs.util.plot_group_stats.

### HLA typing and single-marker association testing

We followed the protocol described by Sakaue et al.^89^ Briefly, we used the ACAF PGEN call set of European participants from the All of Us cohort. Sample quality control was performed as in the GWAS. Variant filtering was conducted using PLINK2^90^ with the following options: --maf 0.005 --chr 6 --geno 0.1 --hwe 1e-10 --snps-only just-acgt --min-alleles 2 --max-alleles 2. Variants were lifted over to hg19 using CrossMap, and phasing was performed with EAGLE (v2.4.1).^91^ HLA imputation was carried out using Minimac4 (v4.1.6)^92^ with the publicly available 1KGP HLA panel (https://github.com/immunogenomics/HLA_analyses_tutorial). Single-marker association testing was performed with PLINK2 --glm, adjusting for age, sex, PC1, and PC2 as covariates. We also performed conditioning using PLINK2. The visualization of HLA structure was performed using PyMOL.

### Genotyping using SNP microarray

We genotyped SNPs from individuals collected at Yale University using the Illumina Global Diversity Array. The genotyping was performed at the Keck Microarray Shared Resource core laboratory at Yale University. We performed standard quality control procedures; variant-level filters included genotyping missingness <5%, MAF > 0.05, HWE p > 1×10^−6^. Genetic ancestry was determined with principal component analysis using 1000 Genomes as a reference panel. Samples of European genetic ancestry were imputed using IMPUTE 2, and strand alignment was performed using SHAPEIT. SNPs with INFO > 0.7 and MAF > 0.01 were retained in the final genomic data.

### Mendelian Randomization Analysis

Two-sample Mendelian randomization (MR) was performed to assess potential causal relationships between EBV DNA positivity and autoimmune diseases. Genetic instruments for EBV DNA positivity were derived from the European-ancestry meta-analysis of EBV DNA GWAS. Instruments were selected at genome-wide significance (*p*<5×10^−^) and clumped using PLINK-based LD clumping (*r*^*2*^<0.0001, *window*=1000kb) with the European 1000 Genomes Phase 3 reference panel. SNPs with significant heterogeneity in the exposure GWAS (HetPVal<0.05) were excluded prior to instrument selection. Outcome GWAS summary statistics were obtained from publicly available sources: MS from the International Multiple Sclerosis Genetics Consortium (IMSGC), RA from the European-ancestry GWAS, SLE from published meta-analyses, and ischemic stroke from the MEGASTROKE consortium (used as a negative control). Harmonization of exposure and outcome data was performed using the genal Python package, with palindromic SNPs retained (action=1). When exposure SNPs were not directly available in outcome datasets, proxy SNPs were identified using LD-based searching (r^2^≥0.6, ±10kb window). MR analyses were conducted using the inverse-variance weighted (IVW) method with random effects as the primary analysis. Sensitivity analyses included the Weighted Median method, and MR-PRESSO for outlier detection and correction. MR-PRESSO^53^ was applied when evidence of pleiotropy or heterogeneity was detected (Cochran’s Q p<0.0001, or discordant IVW and Weighted Median estimates). When MR-PRESSO identified significant outliers, results were reported after outlier removal. Causal estimates are reported as odds ratios with 95% confidence intervals. To assess the contribution of the MHC region, instruments were stratified into MHC-only (chromosome 6: 25–34Mb) and non-MHC variants, and MR analyses were performed separately for each subset. Bidirectional MR was performed to test for potential reverse causation, using genetic instruments for MS, RA, and SLE as exposures and EBV DNA as the outcome.

### Polygenic risk scoring

PRSs were constructed using PRScs^54^ a Bayesian continuous shrinkage method that infers posterior effect sizes by integrating GWAS summary statistics with an external LD reference panel. The final SNP set for each PRS was determined by the intersection of GWAS variants with the reference panel, yielding 729,766 variants for the genome-wide scores. For stratified analyses, MHC-only and no-MHC PRSs were derived by respectively retaining or excluding variants within the HLA region on chromosome 6 (chr6:25–34 Mb, GRCh38).

For validation in the independent Yale cohort, we used GWAS summary statistics from the European-ancestry meta-analysis (All of Us + UK Biobank) for EBV DNA positivity (binary trait) and EBV DNA load (quantitative trait). The Yale cohort comprises European-ancestry individuals at increased risk of MS (relatives of MS patients and those with morphologically suggestive brain MRI lesions; N=94; mean age 40.2±14.7 years; 71% female) with measured EBV DNA load by qPCR. PRS were calculated for each participant, then standardized to mean 0 and SD 1. Participants were grouped into three PRS categories based on cohort-specific percentiles (≤10%, 10–90%, and ≥90%). EBV DNA positivity was modeled using Firth bias-reduced logistic regression with the PRS as either a continuous predictor (per SD increase) or categorical variable (percentile groups), adjusting for age, sex, and the first 10 genotype principal components. Ordinal trend tests across PRS categories were obtained from models including an ordinal coding of the three groups. EBV DNA load was analyzed using linear regression of log10-transformed EBV DNA copies/mL with the same covariate sets.

For the UK Biobank MS association analysis, we used GWAS summary statistics from the European-ancestry subset of All of Us only (to avoid sample overlap) to construct PRSs that were then applied to European-ancestry individuals in the UK Biobank (N=487,181; 2,423 MS cases; 484,758 controls). PRS values were standardized and participants were grouped by percentiles (≤10%, 10–90%, and ≥90%, or quintiles). Associations with MS were assessed using logistic regression adjusted for age, sex, and the first 10 principal components.

### EBV detection by qPCR

Genomic DNA was extracted from whole blood (1–2mL) using the QIAamp DNA Blood Midi kit (Qiagen, 51183), following the manufacturer’s spin-column protocol. Briefly, blood was incubated with a protease and lysis buffer at 70°C for 10 minutes, mixed with 100% ethanol, and loaded onto the spin column. After centrifugation, columns were washed with AW1 and AW2 buffers, and DNA was eluted with 300μL of elution buffer. DNA was stored at −80 °C before analysis.

EBV genomic DNA was quantified by TaqMan qPCR targeting *BamH1W*. Reactions contained DNA (5μL), Fast Advanced qPCR master mix (Applied Biosystems, 444457), primers, and a FAM-labeled probe. qPCR was performed on a StepOnePlus (Applied Biosystems) with the following cycling conditions: 95 °C for 20 s, followed by 40 cycles of 95 °C for 1 s and 60°C for 20 seconds. An EBV DNA standard (ATCC, VR-3247SD: 6.6×10^5^ copies/μL; Lot: 70063234). Ten-fold serial dilutions of the standard (10^5^ to 10^1^ copies) were prepared and run with each qPCR experiment to define assay sensitivity and ensure plate-to-plate consistency. For each run, a standard curve was generated by plotting Ct values against the log-transformed input copy numbers, followed by calculation of the correlation coefficient (R^2^) from the resulting linear regression.

### Meta-analysis of EBV DNA in patient with MS and controls

We searched PubMed from inception to 2025 using predefined keywords (“multiple sclerosis”, “Epstein–Barr virus”, “PCR”, “viral load”). Only peer-reviewed original research articles that examined EBV DNA in peripheral blood from both MS patients and healthy controls using qPCR were included. Because the sensitivity of EBV DNA detection varies across laboratories and assays, we restricted the analysis to studies in which EBV DNA positivity in either group fell within the range of 5–75 percent to minimize the influence of extreme outliers. When raw counts were not reported, the number of EBV-positive subjects was estimated by multiplying the total sample size by the reported percentage and rounding to the nearest integer. Meta-analysis of odds ratios was performed using the metabin function implemented in the meta package in R.

### Single-cell RNA-seq optimized for EBV detection

PBMCs were isolated from whole blood by density-gradient centrifugation using Lymphoprep (STEMCELL, 18061) and cryopreserved in Bambanker Freezing Media (Bulldog-Bio, BB01) for storage in liquid nitrogen until use. PBMCs were thawed rapidly, and B cells were enriched from PBMCs using a pan-B cell isolation kit (STEMCELL, 19554) or B cell isolation kit (STEMCELL, 19054) according to the manufacturer’s protocols. Enriched B cells were counted and adjusted to a final concentration corresponding to a target loading of 20,000 cells.

Tonsil cells were collected by needle aspiration. Collected samples were centrifuged at 500×g for 5 minutes to remove the supernatant, and the cell pellet was resuspended in 1mL PBS. Total and leukocyte counts were determined using trypan blue and methylene blue, respectively. After counting cells were centrifuged at 500×g for 5 minutes, the supernatant was discarded, and the pellet was cryopreserved in Bambanker Freezing Media for storage in liquid nitrogen until use.

To detect the EBV transcript, a custom EBV-specific primer was added to the cell suspension immediately prior to loading onto the Chromium GEM-X Single Cell 5’ v3 Gene Expression platform (10x Genomics). The primer (sequence: 5′-AAG CAG TGG TAT CAA CGC AGA GTA CAA AAC ATG CGG ACC ACC AGC-3′; 10 μM stock, 1 μL added per sample) was synthesized by Integrated DNA Technologies. Libraries were sequenced on an Illumina NovaSeqXP.

### Single-cell RNAseq analysis

The fastq files are processed with cellranger Sequenced reads were processed using Cell Ranger (v8.0.1) with pre-built reference refdata-gex-GRCh38–2024-A and refdata-cellranger-vdj-GRCh38-alts-ensembl-7.1.0 downloaded at 10x Genomics’s Website. EBV transcripts were detected by VIRTUS3. For the pooled samples, donor assignment was performed using the Vireo pipeline^93^ in Demuxafy.^94^ For each sample, cell barcodes and the aligned BAM file generated by Cell Ranger were used as input to generate pileups of exonic SNPs. SNP pileup was performed using cellsnp-lite with the 1000 Genomes Project–derived genotype reference panel (GRCh38; MAF>0.01), applying filters of minimum allele count (minCOUNT≥20) and minor allele frequency (minMAF≥0.1). To restrict genotyping to the relevant donors, we subset the donor VCF to the SNPs detected in the sample-level pileup using bcftools. Vireo was run with an expected donor number of 8 and enabling ambient RNA modeling (--callAmbientRNAs). The analysis was performed using scanpy 1.10.2.^95^ The embedding and cluster assignment was performed using screfmapping pipeline with pre-defined B cell reference.^6^ Briefly, we performed label transfer using CellTypist^96^ with the model Immune_All_Low. B cell population was extracted based on the predicted cluster. Label transfer and embedding was performed by symphonypy with pre-defined B cell reference (the dataset will be published on Figshare).^97^ BCR were analyzed using airrflow and scirpy.^98,99^ DEG analysis was performed using sc.tl.rank_genes_groups, and pathway analysis was performed ClusterProfiler.^100^ For tonsil data analysis, we used Azimuth^101^ with the tonsil reference dataset.^102^

## Extended Data

## Supplementary Material

Supplement 1 Supplementary Table 1 PheWAS of Whole-Blood EBV Positivity in AoU European Participants
Supplementary Table 2 PheWAS of Whole-Blood EBV DNA Load in AoU European Participants
Supplementary Table 3 Number individuals and *λ*_GC_ of individual GWAS
Supplementary Table 4 Lead SNPs of cross-ancestry meta-analysis
Supplementary Table 5 Statistics of EBV Positivity GWAS top hits in all single GWAS
Supplementary Table 6 Statistics of EBV viral load GWAS top hits in all single GWAS
Supplementary Table 7 MAGMA Gene-Set Associations with EBV DNA Positivity
Supplementary Table 8 MAGMA Gene-Set Associations with EBV DNA Load
Supplementary Table 9 HLA allele associated with EBV Positivity
Supplementary Table 10 HLA allele associated with EBV DNA Load
Supplementary Table 11 HLA amino acids associated with EBV Positivity
Supplementary Table 12 HLA amino acids associated with EBV DNA Load
Supplementary Table 13 meta analysis of EBV DNA Positivity
Supplementary Table 14 Sample information for scRNAseq
Supplementary Table 15 Differentially expressed genes in EBV–positive cells
Supplementary Table 16 pathway enriched in EBV–positive cells

## Data Availability

GWAS summary statistics will be available at the GWAS Catalog and PGS Catalog upon acceptance. Locusplot for all lead SNPs and the adjacent region is available at 10.6084/m9.figshare.30712496. Single-cell RNAseq data generated in this study will be deposited in GEO and CZ CELLxGENE upon acceptance. Code generated for this work will be publicly accessible on GitHub upon acceptance. The pipeline used for EBV DNA detection from WGS is available at https://github.com/shohei-kojima/WGS_EBV_search. VIRTUS3 is available at https://github.com/yyoshiaki/VIRTUS3. All codes used for the analysis will be available on github (https://github.com/yyoshiaki/EBV_MS_2025_Manuscript) upon acceptance.
